# CircUBA2 promotes the cancer stem cell-like properties of gastric cancer through upregulating STC1 via sponging miR-144-5p

**DOI:** 10.1186/s12935-024-03423-0

**Published:** 2024-08-05

**Authors:** Jia-Bin Wang, Tong-Xing Lin, Deng-Hui Fan, You-Xin Gao, Yu-Jing Chen, Yu-Kai Wu, Kai-Xiang Xu, Qing-zhu Qiu, Ping Li, Jian-Wei Xie, Jian-Xian Lin, Qi-Yue Chen, Long-Long Cao, Chang-Ming Huang, Chao-Hui Zheng

**Affiliations:** 1 Department of Gastric Surgery, Fujian Medical University Union Hospital, No.29 Xinquan Road, Fuzhou, 350001 Fujian Province China; 2 Fujian Province Minimally Invasive Medical Center, Fuzhou, China; 3 Key Laboratory of Ministry of Education of Gastrointestinal Cancer, Fujian Medical University, Fuzhou, China

**Keywords:** CircUBA2, Gastric cancer, MiR-144-5p, STC1, Stem cell

## Abstract

**Background:**

Cancer stem cells (CSCs) are critical factors that limit the effectiveness of gastric cancer (GC) therapy. Circular RNAs (circRNAs) are confirmed as important regulators of many cancers. However, their role in regulating CSC-like properties of GC remains largely unknown. Our study aimed to investigate the role of circUBA2 in CSC maintenance and the underlying mechanisms.

**Methods:**

We identified circUBA2 as an upregulated gene using circRNA microarray analysis. qRT-PCR was used to examine the circUBA2 levels in normal and GC tissues. In vitro and in vivo functional assays were performed to validate the role of circUBA2 in proliferation, migration, metastasis and CSC-like properties of GC cell. The relationship between circUBA2, miR-144-5p and STC1 was characterised using bioinformatics analysis, a dual fluorescence reporter system, FISH, and RIP assays.

**Results:**

CircUBA2 expression was significantly increased in GC tissues, and patients with GC with high circUBA2 expression had a poor prognosis. CircUBA2 enhances CSC-like properties of GC, thereby promoting cell proliferation, migration, and metastasis. Mechanistically, circUBA2 promoted GC malignancy and CSC-like properties by acting as a sponge for miR-144-5p to upregulate STC1 expression and further activate the IL-6/JAK2/STAT3 signaling pathway. More importantly, the ability of circUBA2 to enhance CSC-like properties was inhibited by tocilizumab, a humanised Interleukin-6 receptor (IL-6R) antibody. Thus, circUBA2 knockdown and tocilizumab synergistically inhibited CSC-like properties.

**Conclusions:**

Our study demonstrated the critical role of circUBA2 in regulating CSC-like properties in GC. CircUBA2 may be a promising prognostic biomarker for GC.

**Graphical Abstract:**

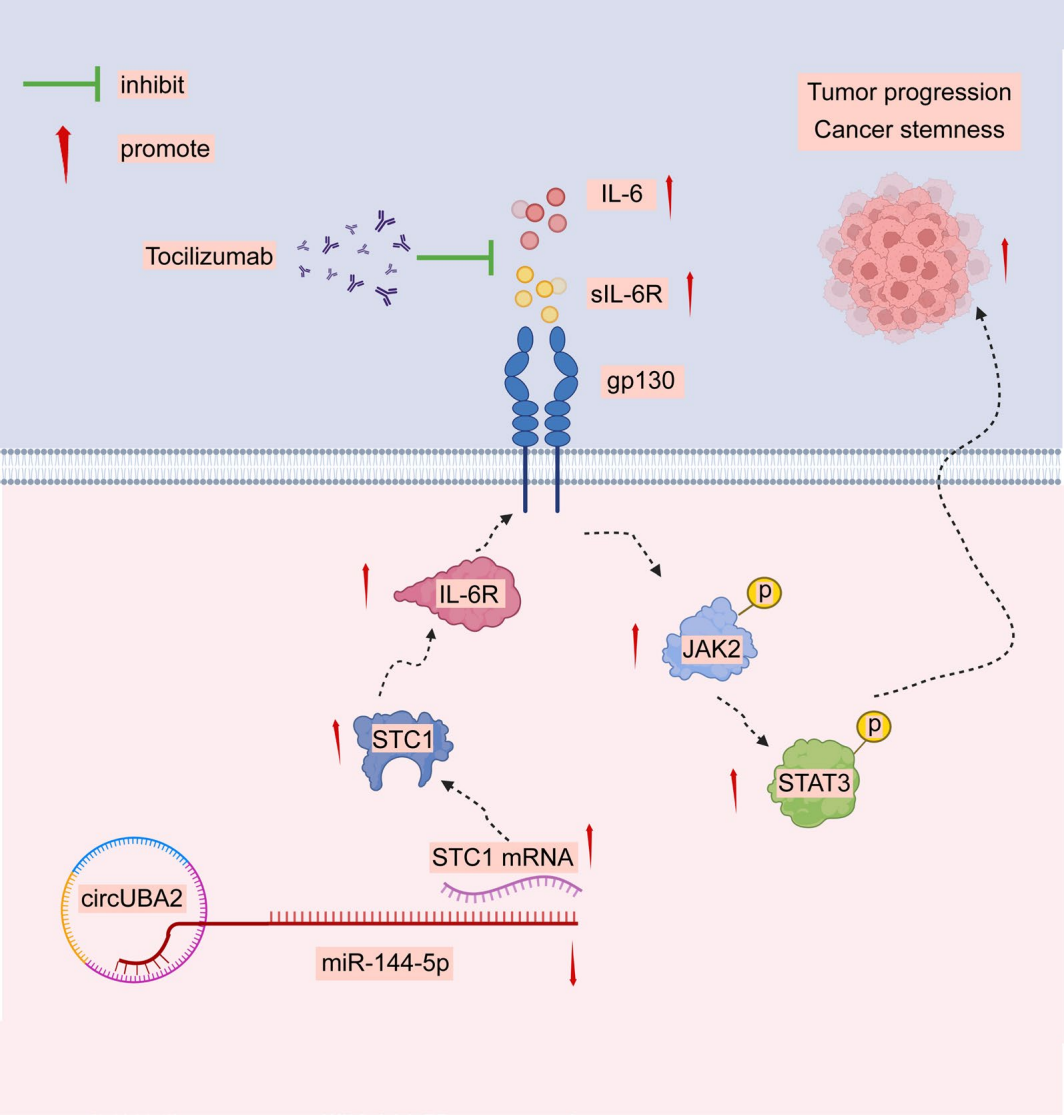

**Supplementary Information:**

The online version contains supplementary material available at 10.1186/s12935-024-03423-0.

## Background

Despite several breakthroughs in the diagnosis and treatment of gastric cancer (GC), its prognosis remains unsatisfactory [1, 2]. Cancer stem cells (CSCs) are one of the most important factors influencing the prognosis of GC and treatment failure [3, 4]. CSCs are a population of cancer cells with the ability to self-renew and initiate tumourigenesis [5], as well as are closely associated with tumour growth, treatment resistance, recurrence, and metastasis. The ineffectiveness of conventional therapies against CSCs necessitates the investigation of new biological markers associated with CSCs survival and their resistance to drugs [6].

CircRNAs are a novel non-coding RNAs, which were with characteristics such as stability, specificity, and highly differential expression abundance [7]. Therefore, circRNAs may have higher accuracy and potential in the diagnosis and prognosis of tumours than other discovered tumour markers [8]. Recently, several differentially expressed circRNAs have been identified in GC tissues, serum, and cells [9, 10]. For example, the expression of circSHKBP1 increased in GC tissues and serum and is associated with a lower survival rate in GC patients [11]. Multiple studies have shown that circRNAs play critical roles in the pathogenesis of GC, including tumour growth, recurrence, and stemness [12–14]. To explore new circRNAs that may serve as diagnostic and prognostic markers for GC, we screened for hsa_cir_0006211, whose expression was significantly upregulated in both the plasma and tissues of GC, based on circRNA microarray analysis and GC tissue expression analysis. We found that hsa_circ_0006211 was spliced from the UBA2 gene, thus, we named this circRNA as circUBA2. However, the biological role of circUBA2 in GC and the associated molecular mechanisms remain unclear.

CircRNAs function in a variety of molecular mechanisms, including serving as sponges for microRNAs (miRNAs) [15–18]. Jin et al. found that the circRNA EPHB4 regulates the proliferation and the stem properties of gliomas by sponging miR-637 and upregulating SOX10 [19]. It has also been demonstrated that circRNAs can regulate CSC-like properties to participate in GC tumourigenesis. For example, CircFAM73A enhanced the CSC-like properties of GC through the miR-490-3p/HMGA2 and β-catenin stabilisation [20]. Moreover, CircSLC4A7 accelerats the stemness and progression of GC by interacting with HSP90 to activate the NOTCH1 signaling pathway [21]. However, the role and specific mechanism of circUBA2 in regulating the CSC-like properties of GC needs to be further clarified.

Previous studies have shown that the IL6/JAK/STAT3 signaling pathway plays an important role in the GC CSC-like properties. For instance, myofibroblasts derived from mouse bone marrow reprogramed non-GC stem cells into CSC-LCs, and then activated the IL-6/STAT3 signaling loop to promote GC metastasis [22]. CircRNAs regulate the CSC-like properties of GC through the IL6/JAK/STAT3 signaling pathway. It has been reported that circFCHO2 promotes proliferation, invasion, angiogenesis, and stem cell properties in GC by activating the JAK1/STAT3 pathway by sponging miR-194-5p [23]. However, there have been no studies on the involvement of circUBA2 in GC CSC-like properties through the IL6/JAK/STAT3 signaling pathway.

Therefore, we investigated whether circUBA2 participates in the development of CSC-like properties in GC. We demonstrated that circUBA2, acting as a sponge for miR-144-5p, upregulated STC1 expression. As a result, the IL-6/JAK2/STAT3 signaling pathway was activated to promote the CSC-like properties, thereby promoting cellular malignancy in GC. Furthermore, elevated circUBA2 levels indicate a poor prognosis for patients with GC. Our study revealed the molecular mechanism by which circUBA2 promotes CSC-like properties of GC and suggested that circUBA2 may become a prospective target for GC therapy.

## Materials and methods

### CircRNA microarray analysis

Three pairs of plasma exon specimens were analysed. The screening condition for differential circRNAs was a fold change of ≥ 2, p < 0.05. Microarray hybridisation and sequencing data acquisition were performed by Kangchen Biotechnology (Shanghai, China). Fifteen circRNAs with the greatest upregulation were selected for cluster analysis, using the analysis software was the R software. (version 4.0.0).

### Human gastric tumour tissues

A total of 168 pairs of matched gastric tumour tissues were collected from the Fujian Medical University Union Hospital between 2014–2016. All gastric tumour samples and paired normal samples were subjected to qRT-PCR analysis. Table 1 lists the clinical characteristics of the patients. The inclusion criteria were as follows: (1) pathological diagnosis as GC; (2) complete clinicopathological features and five years follow-up information; (3) non-detection of distant metastases and other malignancies, and, (4) TNM staging strictly following the 2010 UICC guidelines. Informed consent was obtained from all patients. This study was reviewed and approved by the Ethics Committee of Fujian Medical University Union Hospital (No. 2020KY017).

**Table 1:** Relationship between CircUBA2 expression and baseline characteristics of patients

Variables	Total	CircUBA2 expression		
	(n = 168)	High (%) n = 84	Low (%) n = 84	p value
Gender				0.169
Male	121	65 (77.4)	56 (66.7)	
Female	47	19 (22.6)	28 (33.3)	
Age at surgery (years)				0.737
≤ 65	117	60 (71.4)	57 (67.9)	
> 65	51	24 (28.6)	27 (32.1)	
BMI				0.084
≤ 25	134	62 (73.8)	72 (85.7)	
> 25	34	22 (26.2)	12 (14.3)	
Tumour size (cm)				0.001*
≤ 5	115	68 (81.0)	47 (56.0)	
> 5	53	16 (19.0)	37 (44.0)	
Location of primary tumour				0.922
Lower 1/3	59	30 (35.7)	29 (34.5)	
Middle 1/3	57	27 (32.2)	30 (35.7)	
Upper 1/3	37	20 (23.8)	17 (20.3)	
More than 1/3	15	7 (8.3)	8 (9.5)	
Differentiation grade				0.324
Poor	113	53 (63.1)	60 (71.4)	
Good	55	31 (36.9)	24 (28.6)	
T classification				0.03*
T1	26	17 (20.2)	9 (10.7)	
T2	18	13 (15.5)	5 (6.0)	
T3	70	33 (39.3)	37 (44.0)	
T4	54	21 (25.0)	33 (39.3)	
N classification				0.03*
N0	47	29 (34.5)	18 (21.4)	
N1	26	17 (20.2)	9 (10.7)	
N2	38	16 (19.1)	22 (26.2)	
N3	57	22 (26.2)	35 (41.7)	
M classification				NA
M0	168	84 (100.0)	84 (100.0)	
TNM stage				0.004*
I	28	20 (23.8)	8 (9.5)	
II	40	29 (34.5)	21 (25.0)	
III	90	35 (41.7)	55 (65.5)	

*p < 0.05 was considered significant

### Actinomycin D and RNase R treatment assay

GC cells were intervened with DMSO (Sigma) or actinomycin D at 2.0 μg/mL (Sigma) to block transcription with 6, 12, 18 and 24 h. According to the instructions of the RNeasy MinElute Cleanup Kit (Qiagen), 4ug of RNA was interfered with in 12U of RNase R under experimental conditions of 20 min at 37 °C. The degradation of UBA2 mRNA and circUBA2 in the treated cells was investigated using qRT-PCR.

### Fluorescence in situ hybridization (FISH)

CircUBA2 probe and miR-144-5p probe were both purchased from Service Bio (Wuhan, China). A Nikon Eclipse CI (Nikon, Japan) was used for photography. CircUBA2 was labelled with CY3 and miR-144-5p using FAM. The specific sequence is as follows: circUBA2: 5′-TTCCTTGGCAACCTGTGGAGGTGGAGGG-3′, miR-144-5p: 5′-CTTACAGTATATGATGATATCC-3′.

### RNA immunoprecipitation (RIP)

BGC823 and AGS cells transiently overexpressing either miR-144-5p or miR-NC were collected after 48 h of culturing. The cells were quantified, and 1 × 10^7^ cells were used for the assay. Next, the cells were lysed using a specific lysis solution and incubated for 24 h with a human Ago2 antibody or mouse IgG antibody (Millipore, USA). The RNA/beads complex was resuspended in the buffer and washed repeatedly. Enrichment of the final immunoprecipitated RNA was examined by qRT-PCR. All assays were performed in triplicates, following the protocols of the Magna RIP™ RNA Binding Protein Immunoprecipitation Kit (Millipore, USA).

### Dual-luciferase reporter assay

The plasmids for circUBA2-WT, circUBA2-MUT, STC1-WT and STC1-MUT were designed and constructed by HanBio (Shanghai, China). These plasmids and miR-144-5p mimics were co-transfected into 24-well plates containing BGC823 or AGS cells, respectively, and cultured for 2 d. Cells were collected for full lysis and transferred to separate EP tubes. Luciferase assay reagent II and Stop&Glo II assay reagents were prepared, and a Dual-Luciferase Reporter Assay System (Promega, USA) was used for final fluorescence detection.

### Sphere formation assays

BGC823 or AGS control and treated cells were counted and cultured into ultra-low attachment 6-well dishes (Corning, USA) at a final density of 5000 cells/well. The cells were then incubated in a 5% CO2 incubator at 37 °C for 10 d. The medium used was serum-free DMEM/F12, supplemented with 2% B-27 (Life Technologies, USA), 2 mM L-glutamine (Life Technologies, USA), 20 ng/mL epidermal growth factor (EGF), and 10 ng/mL basic fibroblast growth factor (bFGF). After ten days, the diameter of the spheres was measured and the number of spheres with a diameter > 50 μm was recorded.

### Human GC organoid culture

Fresh GC tissue was digested for 30 min with 2.5 mg/ml Collagenase A (Sigma). A buffer containing sucrose (Sigma) and d-sorbitol (Sigma) was added to the samples with shaking for 2 min. The liquid was passed via a 70 um pore size filter followed by centrifugation at 200 g for 5 min. After washing with PBS, the remaining liquid was then resuspended in Matrigel (Corning). Next, 50 µL of Matrigel suspension was slowly added to each well of a 24-well plate and 500 µL Organoid Growth Medium (StemCell Technologies, Cambridge, MA) was added for organoid growth. The organoids were grown in a 5% CO2 incubator (Thermo Fisher Scientific, USA) at 37 °C and the medium was changed every three days in order to ensure the nutrition of the organoids. Second-generation organoids were infected with circUBA2 lentivirus or treated with drugs, and the organoids were measured and analysed seven days later.

### Tumour formation and metastasis assays

This study involved a nude mouse xenograft tumour model, a lung metastasis model, a liver-spleen metastasis model, and an in vivo limiting dilution assay. Four-week-old male nude BALB/c mice (male) were used. The xenograft tumour model was created by injecting 5 × 10^6^ stably transfected BGC823 cells or control cells into the axillae of nude mice subcutaneously, and 3–4 weeks later the mice were euthanised and the weight of the generated tumour was measured. Tumour size was measured every three days during this period and was calculated as V = (L × W^2^)/2 mm^3^ (V, volume; L, length; W, width). Cells (5 × 10^6^) were injected into the tail vein or spleen of nude mice to establish lung or liver metastasis models. Four weeks later, the lungs or livers of the mice were removed, metastases were recorded, and specimens were carefully preserved. For the *vivo* limiting dilution assay, different numbers (1 × 10^5^, 1 × 10^4^, 5 × 10^3^, 1 × 10^3^) of stably transfected BGC823 cells or control cells were injected into the subcutaneous tissue of nude mice.

The tumours were removed after four weeks, and the number of tumours was counted and analysed. This study was reviewed and approved by the Ethics Committee of Fujian Medical University/Laboratory Animal Centre (Fuzhou, China) (ethical approval number of animal experiments: FJMU IACUC 2021-0047).

### Cytokine array analysis

The cells were seeded in serum-free RPMI on 10 cm plates for 24 h at a cell concentration of 1 × 10^6^ cells per well. The cell medium was collected into EP tubes and centrifuged at 800 × *g* for 20 min at 4 °C to obtain the supernatants for subsequent analysis. Analysis was performed according to the manufacturer’s instructions for human cytokine antibody array membranes (ab133997, Abcam, UK).

### Statistical analysis

All data were analysed using the R software (version 4.0.0; https://www.R-project.org/) and the SPSS software (version 25.0; IBM Corporation, New York, USA). Normality tests were used to determine the data, using parametric or non-parametric tests. Continuous variables with normally distribution and variance homogeneity were analysed using an independent-sample t-test or analysis of variance. LSD-t test is used for pairwise comparison between groups. A non-parametric test was used for non-normal distributions or uneven variance. The chi-square test was used to examine the association between circUBA2 expression and clinicopathological parameters. Overall survival analysis was performed using Kaplan–Meier analysis, and the log-rank test was used to compare differences. Spearman’s rank analysis was performed to determine the correlation between circRNA, miRNA or mRNA expression levels. p < 0.05 was considered to be statistically significant.

Other details of the experiments involved in this study are provided in Additional File 1 and Additional File 2.

## Results

### CircUBA2 is upregulated in GC and associated with poor prognosis

**Fig. 1:**
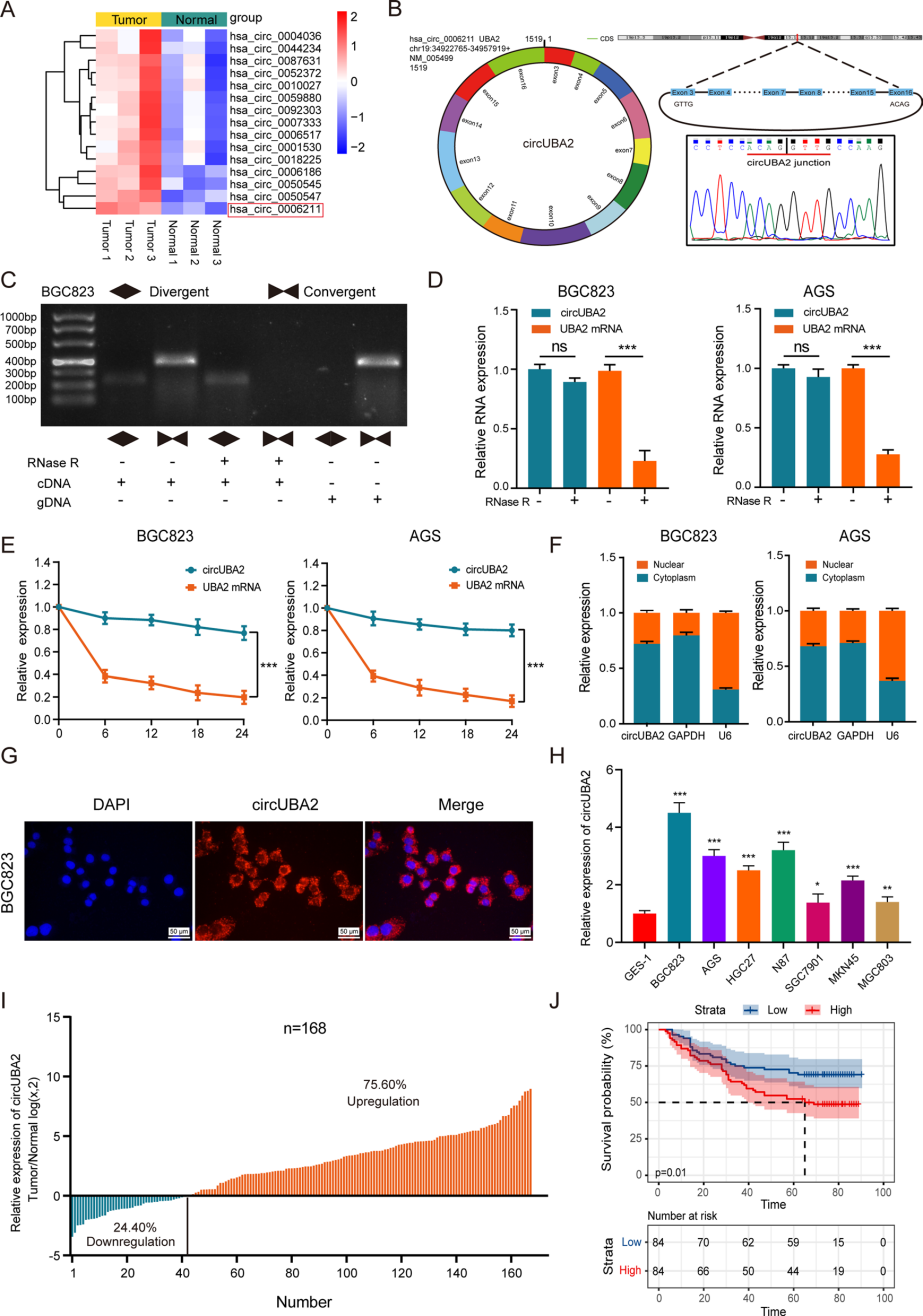
The expression, identification and prognostic significance of circUBA2 in GC. **A** Clustered heatmap of the top 15 differentially expressed circRNAs between 3 pairs of plasma specimens. **B** CircUBA2 (hsa_circ_0006211) is generated from the UBA2 gene that is located on chromosome 19, and the “head-to-tail” splicing sites of circUBA2 were confirmed by Sanger sequencing. **C** The presence of circUBA2 in BGC823 cells is verified by gel electrophoresis experiments. **D** qRT-PCR determining the abundance of circUBA2 and linear UBA2 in the BGC823 and AGS cell lines after RNase R treatment. **E** qRT-PCR determining the abundance of circUBA2 and linear UBA2 in the BGC823 and AGS cell lines after actinomycin D treatment. **F** Nucleoplasmic separation assays for the relative expression of circUBA2 in BGC823 and AGS cells. **G** FISH for circUBA2 localization in BGC823 cell. **H** The expression of circUBA2 in seven GC cell lines and normal gastric epithelial cells (GES-1) was quantified by qRT-PCR. **I** The T/N ratio of mRNA expression levels of circUBA2 in 168 paired GC samples after log-transformation (log 2). **J** The Kaplan–Meier method was used to analyse the overall survival rate of 168 patients. 168 patients were divided into two groups based on the median level of circUBA2 expression. All data were presented as mean ± SD. *p < 0.05; **p < 0.01; ***p < 0.001

To define the function of circRNAs in the pathogenesis of GC, we collected preoperative plasma samples from three healthy individuals and three patients with GC to perform circRNA microarray analysis. Hierarchical cluster analysis revealed that hsa_circ_0006211 was the most upregulated circRNA in those plasma of patients with GC (Fig. 1A). We further examined the expression of these 15 circRNAs in 60 pairs of GC tumour tissues as well as in matched normal tissues and found that only hsa_cir_0006211 and hsa_cir_0050545 were expressed at higher levels in GC tissues than in paired adjacent normal tissues, and the difference in hsa_cir_0006211 expression was more significant (Fig. S1A). This indicated that hsa_circ_0006211 may play a critical role in GC progression, which was why we selected hsa_circ_0006211 for subsequent studies. We found that hsa_circ_0006211 was spliced from the UBA2 gene positioned at chr19:34,922,765–34,957,919 and formed a loop transcript of 1519 nt, thus, we named this circRNA as circUBA2. Sanger sequencing was performed to identify the splice site of circUBA2 (Fig. 1B). To determine whether the head-to-tail splicing of circUBA2 originates from trans-splicing or genomic rearrangements, we designed two types of primers, diverging primers for circUBA2 amplification and convergent primers for linear UBA2 mRNA amplification, and then extracted gDNA and cDNA from BGC823 cells. The results of gel electrophoresis revealed that circUBA2 was not detected in gDNA, but was detected in cDNA, showing that the specific circular structure of circUBA2 was indeed formed by reverse splicing (Fig. 1C). BGC823 and AGS cells were treated with RNase R to assess the stability of circUBA2. The results revealed that circUBA2 resisted RNase R digestion, whereas the linear structure of UBA2 is significantly degraded (Fig. 1D). The resistance of cirUBA2 to actinomycin D digestion further confirmed its loop structure (Fig. 1E). In addition, nuclear-cytoplasmic isolation and FISH assays illustrated that circUBA2 was predominantly localised in the cytoplasm (Fig. 1F, G). qRT-PCR analysis was conducted to assess the abundance of circUBA2 in eight GC cell lines (Fig. 1H). To assess the influence of cirUBA2 on GC, we examined its expression in 168 pairs of GC tumour tissues and matched normal tissues. The experiments revealed that compared to the corresponding adjacent normal tissues, GC tissues showed overexpression of circUBA2 in 75.60% (127/168) of tissues from the same patients (Fig. 1I). To further analyse the correlation between circUBA2 levels and clinicopathological characteristics, the tissues were split into two groups, a high circUBA2 group and a low circUBA2 group, according to the median circUBA2 expression. Analysis of pathological features suggested that circUBA2 expression was significantly correlated with tumour size, T stage, N stage, and TNM stage (Table 1). Furthermore, Kaplan–Meier analysis clearly illustrated that the survival rate of GC patients with high circUBA2 expression was lower than that of patients with low circUBA2 expression (p = 0.01) (Fig. 1J). In conclusion, circUBA2 was identified as a highly stable circRNA and a novel diagnostic and prognostic marker for GC.

### CircUBA2 promotes the proliferation, migration and metastasis of GC cells

**Fig. 2:**
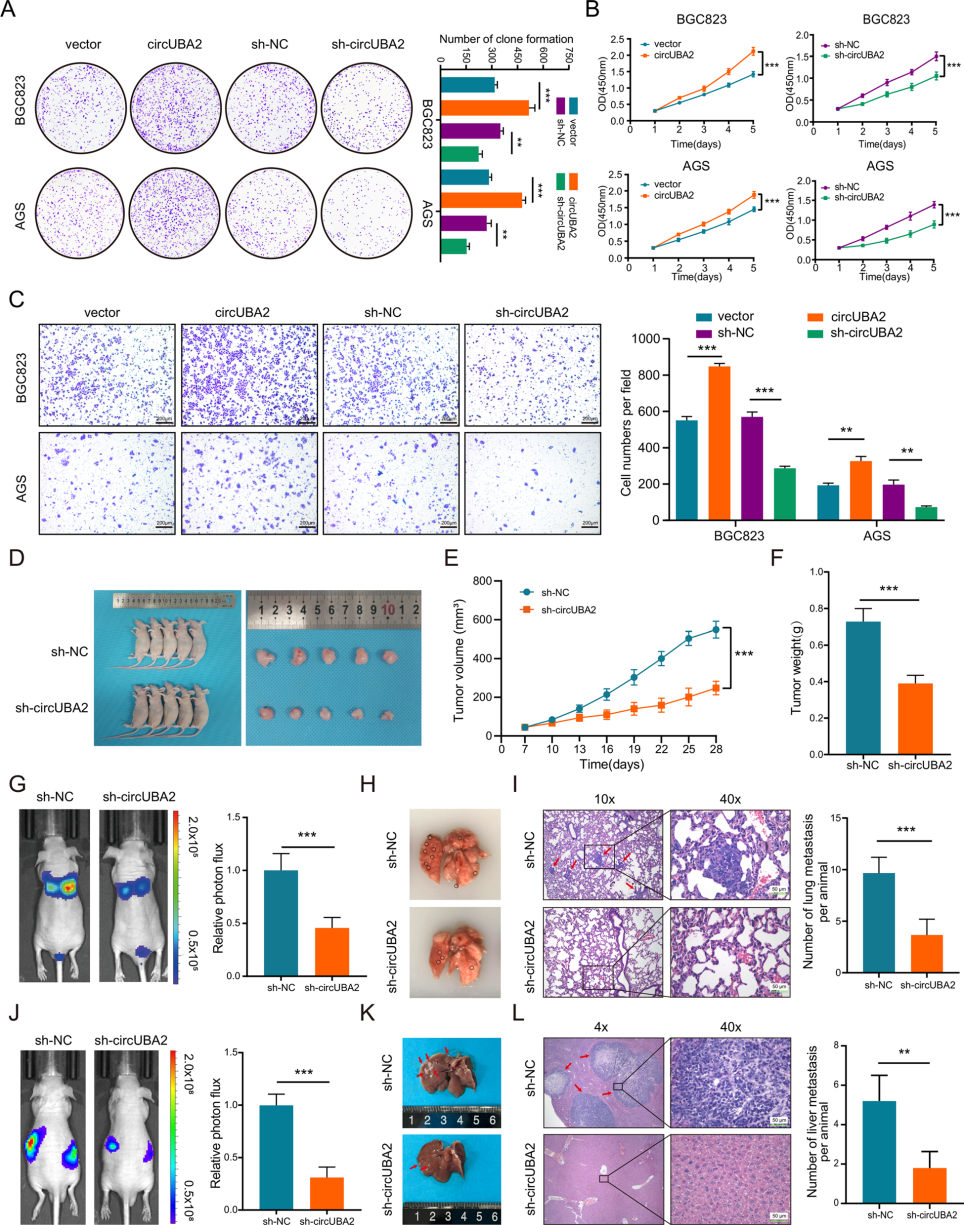
CircUBA2 promotes the proliferation, migration, and metastasis of GC cells. **A**, **B** Colony formation and CCK-8 assays were performed in BGC823 and AGS cells transfected with the vector, circUBA2, control or circUBA2 shRNA. **C** A transwell assay was performed to evaluate the migratory capacity of BGC823 and AGS cells, scale bar = 200 μm. **D** Images of nude mice and tumours from the sh-NC and sh-circUBA2 groups (n = 5 mice per group). **E** Growth curves of xenograft tumours after 4 weeks. **F** Weight of the xenograft tumours after 4 weeks. **G** Representative images for in vivo images of lung metastasis models and quantitative fluorescence results (n = 5 mice per group). **H**, **I** Representative images and quantitative results of lung metastases and H&E staining in the sh-NC and sh-circUBA2 groups, scale bar = 50 μm. **J** Representative in vivo images of liver metastasis models and quantitative fluorescence results (n = 5 mice per group). **K**, **L** Representative images and quantitative results of liver metastases and H&E staining in the sh-NC and sh-circUBA2 groups, scale bar = 50 μm. *p < 0.05; **p < 0.01; ***p < 0.001

To evaluate whether circUBA2 influences the function of GC cells, we transfected overexpressed circUBA2 or sh-circUBA2 lentiviruses into BGC823 and AGS cells and verified their efficiency by qRT-PCR. The results demonstrated a corresponding change in the expression of circUBA2, whereas the change in the expression of UBA2 was not statistically significant, confirming that BGC823 and AGS cells with steady overexpression or downregulation of circUBA2 were successfully generated (Fig. S1B). We investigated the effect of circUBA2 on cell proliferation using colony formation and CCK-8 assays. We found that the downregulation of circUBA2 inhibited the proliferation of BGC823 and AGS cells, whereas the overexpression of circUBA2 promoted cell proliferation (Fig. 2A, B). Flow cytometry assessment of the cell cycle revealed that downregulation of circUBA2 increased the proportion of cells in the G0/G1 phase but reduced the proportion of cells in the S phase in both BGC823 and AGS cells, whereas overexpression of circUBA2 resulted in the opposite trend (Fig. S1D). We further examined the expression of cell cycle proteins related to the G1/S transition, and the results suggested that downregulation of circUBA2 decreased the expression of cyclin D1, cyclin E1, and CDK2. By contrast, circUBA2 overexpression increased the expression of these proteins (Fig. S1C). These results showed that downregulation of circUBA2 resulted in cell cycle stagnation in the G0/G1 phase, resulting the in repression of GC cell proliferation. We also found that the downregulation of circUBA2 inhibited cell migration in vitro. Conversely, the upregulation of circUBA2 expression resulted in significantly enhanced mobility (Fig. 2C).

**Fig. 3:**
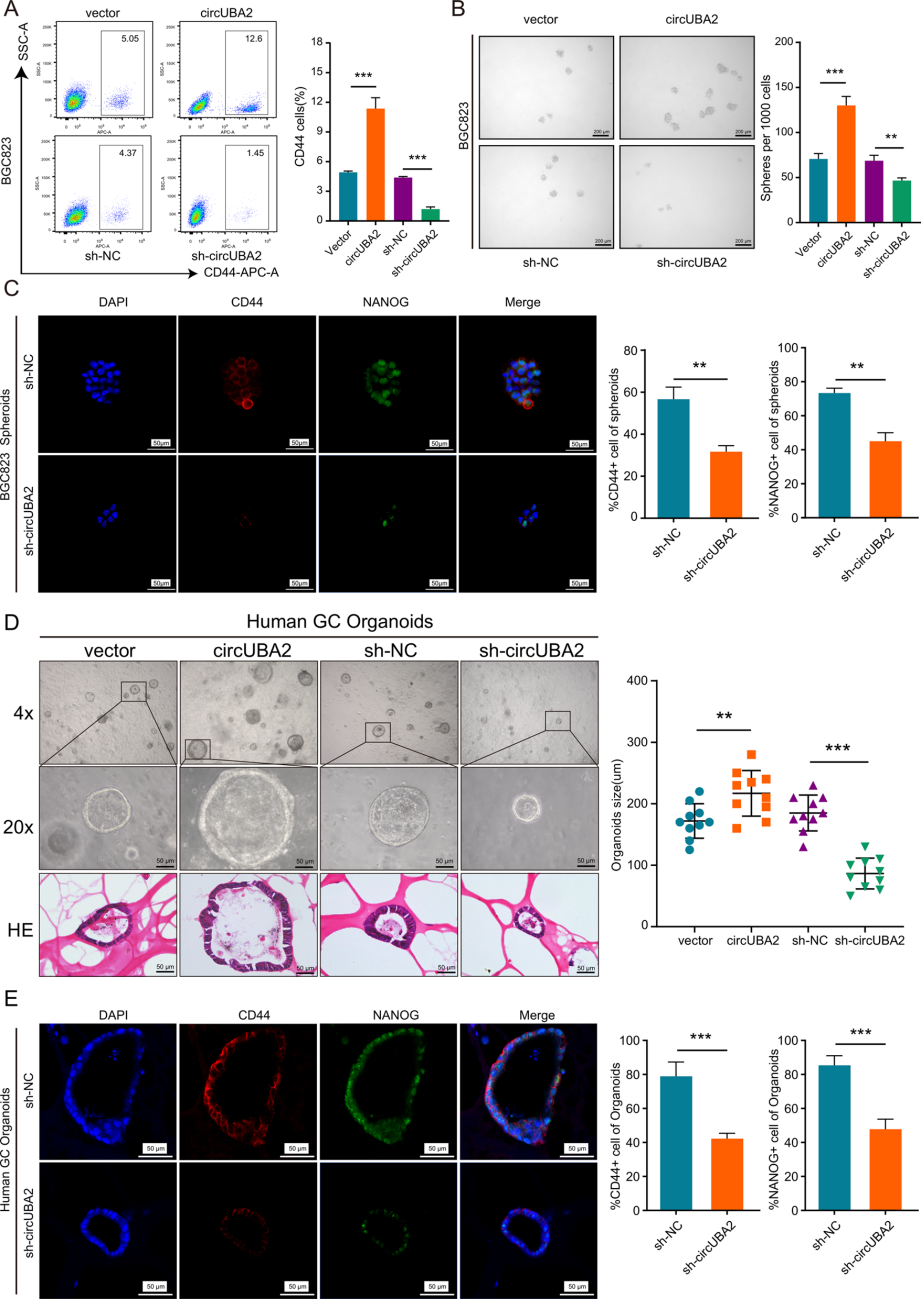
CircUBA2 enhances the stem cell-like properties of GC cells. **A** Proportion of CD44-positive cells and statistical results for BGC823 cells. **B** Representative images and statistical result of sphere formation assay in BGC823 cells, scale bar = 200 μm. **C** IF staining for CD44 (red), NANOG (green), and DAPI (blue) in BGC823 cells. The percentages of CD44 and NANOG staining were quantified, scale bars = 50 μm. **D** GC tissues of human organoids were stably transfected with the vector, circUBA2, control, or sh-circUBA2 lentivirus, showing that overexpression of circUBA2 promoted organoid growth, whereas circUBA2 knockdown inhibited organoid growth. **E** IF staining for CD44 (red), NANOG (green), DAPI (blue) in human GC organoids. The percentages of CD44 and NANOG staining results were quantified, scale bars = 50 μm. *p < 0.05; **p < 0.01; ***p < 0.001

We explored the role of circUBA2 in GC cells in vivo and constructed a subcutaneous xenograft tumour model. After knocking down circUBA2, tumour weight was substantially reduced and the tumour growth rate was significantly lower compared to tumours in the sh-NC group (Fig. 2D–F). After the overexpression of circUBA2, these results were reversed (Fig. S1E-G). We successfully constructed a model of lung metastasis. Four weeks after injection, lung metastases were significantly reduced in the circUBA2 knockdown group of mice, as reflected by fluorescence imaging (Fig. 2G). Observation of the excised lung tissue revealed that the circUBA2 knockout group had fewer lung metastases than the control group (Fig. 3H). This result was supported by the H&E staining of the resected lung tissue (Fig. 3I). Furthermore, a liver-spleen metastasis model was established, which revealed that the downregulation of circUBA2 inhibited the liver metastasis capacity of BGC823 cells (Fig. 2J–L).

In summary, circUBA2 increased the proliferation, migration, and metastasis of GC cells.

### CircUBA2 elevates stem cell-like properties in GC cells

Acquisition of cancer stem cell-like properties has been reported to be strongly related to the development, maintenance, and metastasis of GC [4]. To explore whether circUBA2 influenced the biological features of GC cells through the acquisition of CSC-like properties, we first investigated the correlation between circUBA2 expression and stem cell-like markers. qRT-PCR showed that circUBA2 increased the expression of CD44, NANOG, SOX2 and SOX9, but had no correlation with CD24, LGR5, OCT4, and CD133. These results were confirmed using western blotting (Fig. S2A-B). Flow cytometry indicated that the ratio of CD44-positive BGC823 cells to AGS cells decreased after circUBA2 knockdown, whereas these results were reversed when circUBA2 was overexpressed (Fig. 3A, Fig. S2C). Moreover, circUBA2 overexpression promoted the generation of human GC organoids and tumour spheres in BGC823 and AGS cells, respectively. This effect was reversed by circUBA2 knockdown (Fig. 3B, D; Fig. S2D). The transfection efficiency of circUBA2 overexpression or sh-circUBA2 in human GC organoids was verified by qRT-PCR (Fig. S2E). The percentage of CD44-NANOG-positive cells increased in the circUBA2 group but decreased in the inhibition group of circUBA2 (Fig. 3C, E, Fig. S3). We also confirmed the involvement of in the induction of self-renewal using limiting dilution assays in vitro and in vivo (Fig. S2F–K). Moreover, Immunohistochemical (IHC) staining assays of subcutaneous tumours in nude mice showed that the percentage of stem cell-like marker-positive cells was significantly higher in the circUBA2 group than in the control group; however, the opposite was observed in the inhibition group of circUBA2 (Fig. S4A–E). This result was confirmed by western blotting of the subcutaneous tumours (Fig. S4F). These observations indicate that circUBA2 enhances the stem cell-like properties of GC cells.

### CircUBA2 affects the expression of STC1 by acting as a sponge for miR-144-5p

**Fig. 4:**
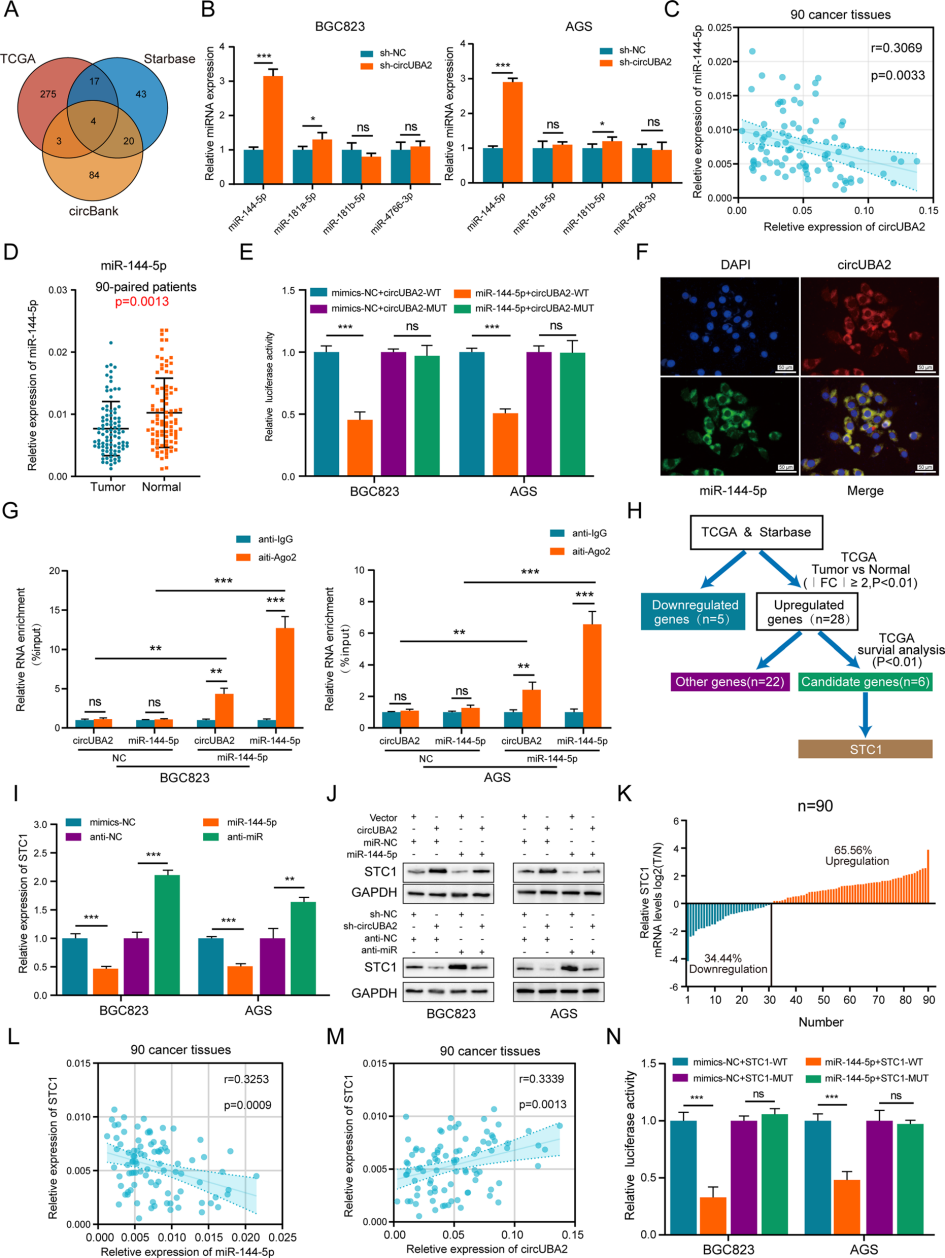
CircUBA2 affects the expression of STC1 by acting as a sponge for miR-144-5p. **A** Venn diagram showing potential miRNAs in TCGA, Starbase, and circBank that bind to circUBA2. **B** Quantification of the expression levels of four overlapping miRNAs in control and circUBA2 knockdown BGC823 and AGS cells by qRT-PCR. **C** Correlation between circUBA2 and miR-144-5p expression in GC samples. **D** The mRNA expression of miR-144-5p in 90-paired GC tissues was determined by qRT-PCR. **E** The targeting relationship between circUBA2 and miR-144-5p in BGC823 and AGS cells was verified by luciferase activity assay. **F** FISH assay detecting the colocalisation of circUBA2 with miR-144-5p in the cytoplasm of BGC823 cells, scale bar = 50 μm. **G** RIP assay was used to detect the enrichment of circUBA2 and miR-144-5p in BGC823 and AGS cells. **H** A flowchart of the screening process for miR-144-5p downstream candidate genes. **I** Determination of mRNA expression levels of STC1 in miR-144-5p mimics-treated or inhibitor-treated and control BGC823 and AGS cells using qRT-PCR. **J** Western blotting of STC1 in BGC823 and AGS cells transfected with the indicated vectors. **K** The T/N ratio of mRNA expression levels of STC1 in 90 paired GC samples after log-transformation (log 2). **L** The correlation between miR-144-5p and STC1 expression in GC samples. **M** The correlation between circUBA2 and STC1 expression in GC samples. **N** The relationship between STC1 and miR-144-5p in BGC823 and AGS cells was verified using a luciferase activity assay. *p < 0.05; **p < 0.01; ***p < 0.001

We first predicted potential differentially expressed target miRNAs using three databases (TCGA, CircBank, and Starbase) and obtained four miRNAs after taking intersections (Fig. 4A). qRT-PCR revealed that only miR-144-5p expression was significantly elevated in circUBA2-downregulated BGC823 and AGS cells (Fig. 4B). Furthemore, the expression of miR-4766-3p and miR-144-5p was downregulated in 90 pairs of GC samples, whereas miR-144-5p and circUBA2 expression was negatively correlated (r = 0.3069, p = 0.0033) (Fig. 4C, D, Fig. S5). Next, we found that luciferase activity was significantly altered in cells co-transfected with the miR-144-5p and circUBA2-wild-type (WT) reporter genes (Fig. 4E). Subsequently, FISH results showed that miR-144-5p and circUBA2 preferentially co-localised in the cytoplasm (Fig. 4F). The results of the anti-AGO2 RIP assay showed that AGO2-induced endogenous circUBA2 pulldown was specifically enriched in both BGC823 and AGS cells after miR-144-5p overexpression (Fig. 4G), confirming the direct binding of circUBA2 to miR-144-5p.

Based on these results, we hypothesised that circUBA2 regulates GC malignancy and stemness by sponging miR-144-5p. To test this hypothesis, we immediately transfected miR-144-5p mimics into circUBA2 overexpressing cells, as well as miR-144-5p inhibitors into circUBA2-silenced BGC823 and AGS cells. The experimental results showed that miR-144-5p mimics impaired circUBA2-mediated promotion of GC cell proliferation and migration. Inhibitors of miR-144-5p reverse the inhibitory effects of circUBA2 suppression. These experiments included colony formation, transwell, and cell cycle assays (Fig. S6-7). Western blotting, sphere formation assays, and flow cytometry of CD44-positive cell also demonstrated that circUBA2 promoted stemness by sponging miR-144-5p (Fig. S8). These results illustrate that circUBA2 acts as a sponge for miR-144-5p, promoting cellular malignancy and cancer stemness in GC cells.

To identify the downstream targets of miR-144-5p, we analysed the TCGA and Starbase databases and screened six candidate genes with upregulated expression in TCGA (Fig. 4H). At the mRNA level, only STC1 expression was downregulated in miR-144-5p mimic-treated GC cells and was upregulated in miR-144-5p inhibitor-treated GC cells, and the expression of HOXA13, COL8A1, BCAT1, PDSS1, MTPAP was not statistically significant (Fig. 4I, Fig. S9A-E). Similar changes were observed in the protein expression level of STC1 (Fig. 4J). Moreover, STC1 expression was higher in 90 pairs of GC tissue samples than in healthy tissue samples, not only at the protein level (Fig. S9F-G) but also mRNA levels (Fig. 4K). The mRNA level of STC1 was visibly negatively correlated with miR-144-5p (Fig. 4L) and positively correlated with circUBA2 (Fig. 4M) in these samples. We also assessed the prognostic profile of STC1 in GC by IHC in another internal cohort. A distinct differencet in STC1 staining was observed between tumour and non-tumour tissue (Fig. S9H-I). STC1 expression was low in 78 (37.14%) samples and high in 132 (62.86%) samples. Combined with the survival information from these patients, the STC1 high expression group showed worse survival (Fig. S9J-K). To confirm whether STC1 has the same expression profile and prognosis in different populations, we evaluated patients using TCGA and obtained similar results (Fig. S9L-N). Rescue experiments showed that STC1 knockdown significantly reversed the promoting effects of the miR-144-5p inhibitor on GC cell proliferation and migration (Fig. S10). Dual-luciferase reporter assays demonstrated the binding of miR-144-5p to STC1 (Fig. 4N).

In summary, these results confirmed that circUBA2 regulates STC1 expression by sponging miR-144-5p.

### CircUBA2 regulates the expression level of STC1 and promotes GC growth, metastasis and CSC-like properties

**Fig. 5:**
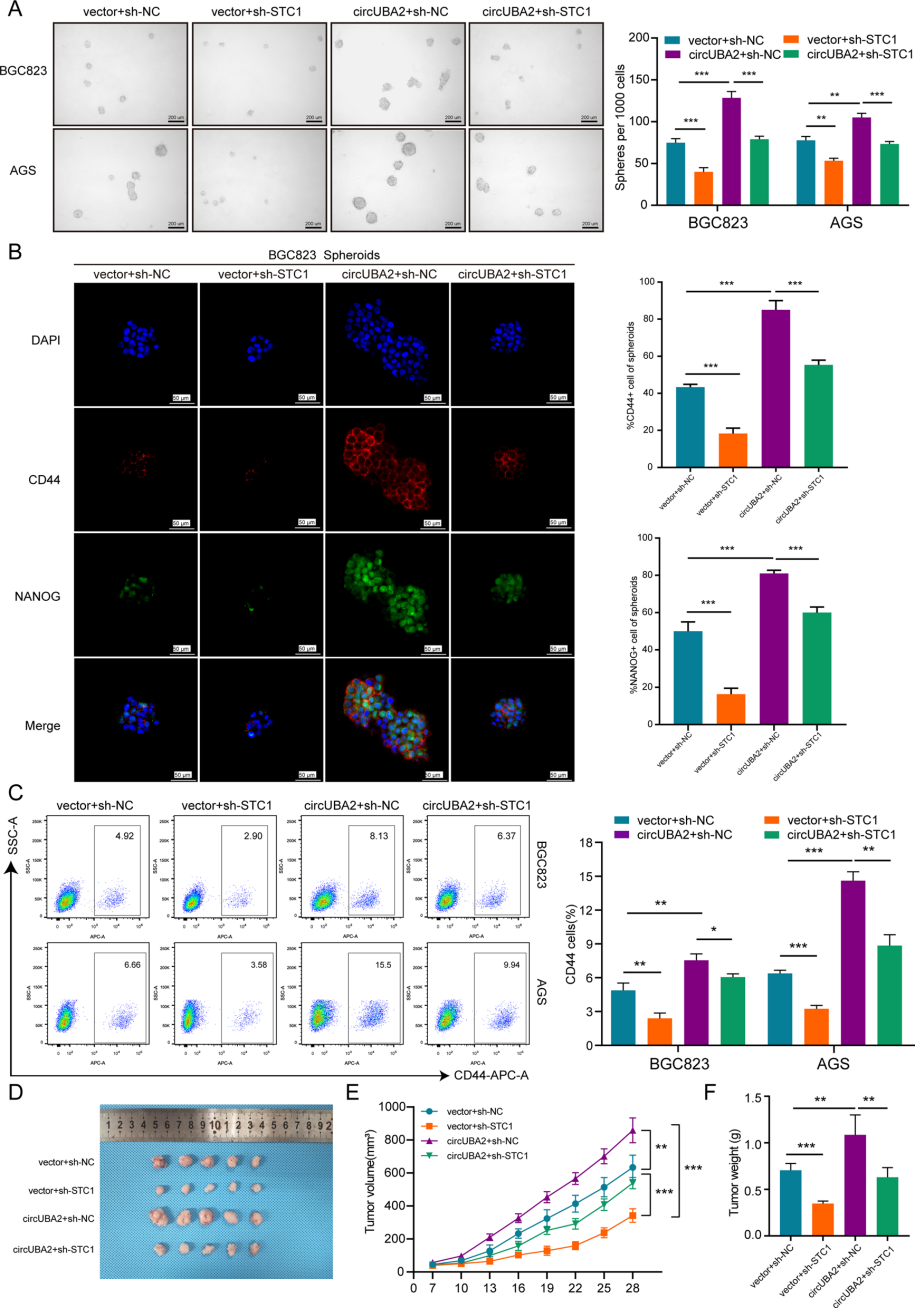
CircUBA2 regulates the expression level of STC1 and promotes GC growth, metastasis, and CSC-like properties. **A** Representative images and statistical results of the sphere formation assay in BGC823 and AGS cells, scale bar = 200 μm. **B** IF staining for CD44 (red), NANOG (green), and DAPI (blue) in BGC823 cells. The percentages of CD44 and NANOG staining results were quantified, scale bars = 50 μm. **C** Proportion of CD44-positive BGC823 and AGS cells and statistical results. **D** Images of xenograft tumours from the four groups (n = 5 mice per group). **E** Growth curves of xenograft tumours after 4 weeks. **F** Weight of the xenograft tumours after 4 weeks. *p < 0.05; **p < 0.01; ***p < 0.001

To determine whether circUBA2 exerted its biological effects by sponging miR-144-5p to affect STC1 expression, we suppressed STC1 expression in circUBA2-overexpressing cells. Sphere formation assays showed that STC1 downregulation decreased the sphere-forming ability of BGC823 and AGS cells, whereas overexpression of circUBA2 in STC1 downregulated cells partially restored this ability (Fig. 5A). Immunofluorescence (IF) experiments revealed a reduction in the percentage of CD44-positive or NANOG-positive cells after STC1 downregulation, and circUBA2 overexpression attenuated the effects of STC1 disruption (Fig. 5B). Similarly, flow cytometry revealed a decreased proportion of CD44-positive cells in the STC1-silenced group, which was reversed by circUBA2 overexpression (Fig. 5C). Based on further cytological assays, including colony formation, transwell assays, and cell cycle assays, circUBA2 overexpression also reversed the suppressive effect of STC1 repression on GC cell proliferation and migration (Fig. S11).

To explore the roles of circUBA2 and STC1 in vivo, we also developed a xenograft tumour model and compared it to the group injected with control cells; xenograft tumours following the injection of STC1-silenced BGC823 cells showed a slower growth rate. However, circUBA2 overexpression partially reversed this effect (Fig. 5D–F). IHC staining of these xenograft tumours for previous stem cell-like markers, compared to the control xenograft tumours, showed that the percentage of CD44, NANOG, SOX9, SOX2 positive cells was reduced in the STC1-silenced group, and STC1 shRNA and circUBA2 co-transfection played a reversal role (Fig. S12). The results from the liver and lung metastasis models clearly indicated that circUBA2 elevated GC metastasis in vivo, similar to the effects of STC1 (Fig. S13).

In summary, circUBA2 regulates the expression level of STC1 and promotes GC growth, metastasis, and CSC-like properties.

### CircUBA2/miR-144-5p/STC1 regulates IL-6 expression and activates JAK2/STAT3 signaling

**Fig. 6:**
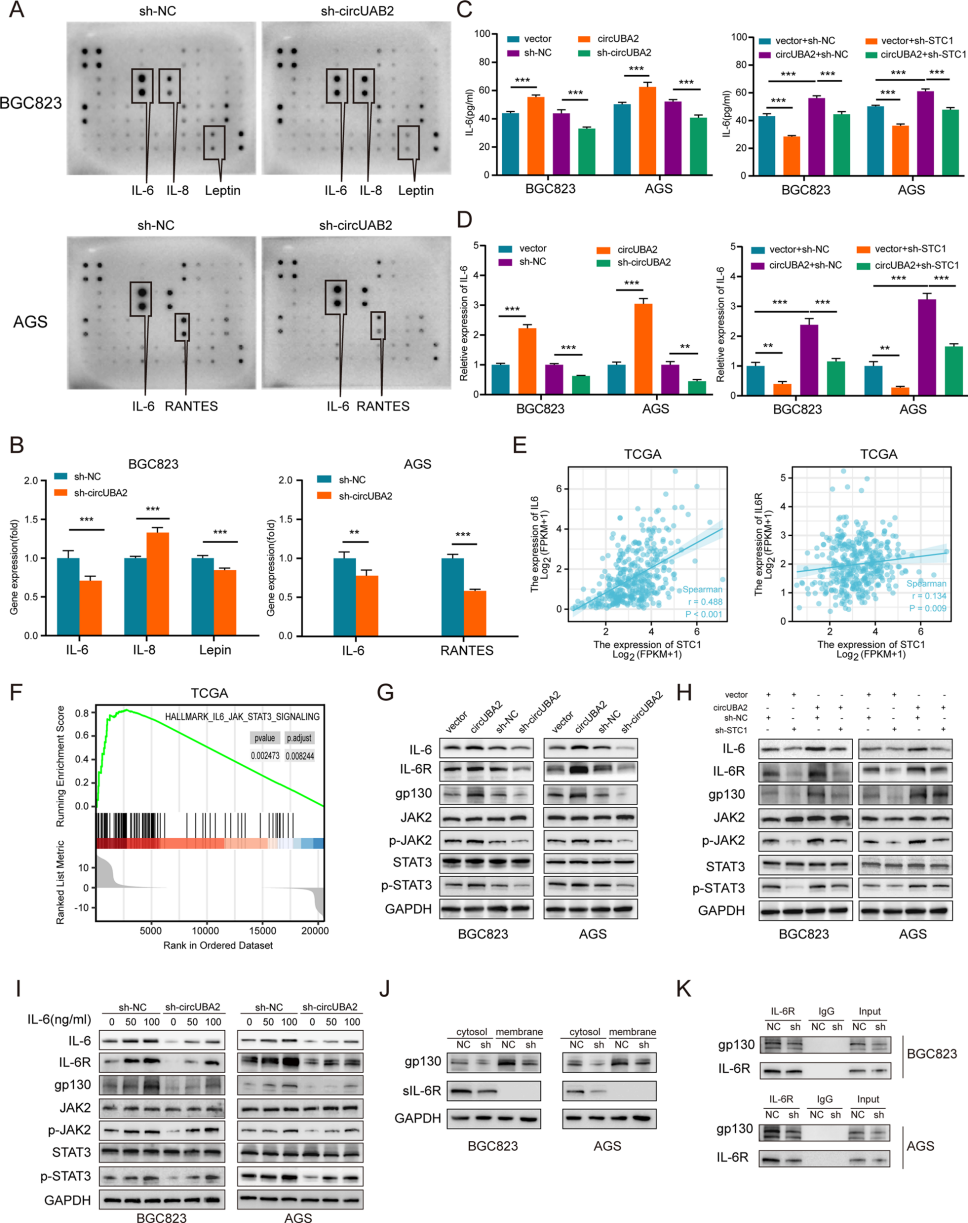
CircUBA2/miR-144-5p/STC1 regulates IL-6 expression and activates JAK2/STAT3 signaling. **A** Cytokine arrays were incubated with the supernatants of BGC823 and AGS cells transfected with knockdown circUBA2 or control lentivirus to identify differential cytokines. **B** Quantification of the experimental results for the cytokine arrays. **C** IL-6 expression in the supernatants of BGC823 and AGS cells was determined by ELISA. **D** Relative expression of IL-6 in stably transfected BGC823 and AGS cells was measured by qRT-PCR. **E** The correlation between STC1 and IL-6 or IL-6R according to TCGA’s GC samples. **F** Gene set enrichment analysis (GSEA) revealed a notable relationship between STC1 and ‘HALLMARK_IL6_JAK_STAT3_SIGNALING’ signaling pathway ( p = 0.002473). **G**, **H** Protein levels of IL-6/JAK2/STAT3 pathway members in stably transfected BGC823 and AGS cells were determined by Western blotting. **I** Protein levels of the IL-6/JAK2/STAT3 pathway members in BGC823 and AGS cells stably transfected with different doses of IL-6 were determined by western blotting. **J** Protein expression of gp130 and sIL-6R in the cell membrane and cytoplasm of sh-NC and sh-circUBA2 groups was detected by western blotting. **K** The interaction between gp130 and IL-6R in BGC823 and AGS cells was identified by co-immunoprecipitation (co-IP). *p < 0.05; **p < 0.01; ***p < 0.001

We investigated the possible mechanisms through which circUBA2 regulates tumour progression and stemness in GC cells. Cytokine arrays were incubated with conditioned media from BGC823 or AGS cells to determine the relevant cytokines in the cancer cells. The results showed that in BGC823 cells, Interleukin-6 (IL-6), and leptin secretion were reduced and IL-8 secretion was increased in circUBA2-silenced cells relative to control cells. In circUBA2-silenced AGS cells, IL-6 and RANTES secretion was reduced (Fig. 6A, B). ELISA using an anti-IL-6 antibody showed that circUBA2 overexpression promoted IL-6 secretion, whereas knockdown of circUBA2 significantly inhibited IL-6 secretion. Moreover, circUBA2 overexpression was able to reverse the effects of sh-STC1 on IL-6 secretion (Fig. 6C). Changes in IL-6 mRNA levels were confirmed by qRT-PCR (Fig. 6D). Similar, results were obtained for the qRT-PCR of IL-6R and the ELISA of soluble IL-6R (sIL-6R) (Fig. S14A-D). In the TCGA database, both IL-6 and IL-6R expression positively correlated with STC1 (Fig. 6E). Based on the TCGA database, we chose gene set enrichment analysis (GSEA) to identify the pathways enriched in STC1 high-expressing tumours compared to those in STC1 low-expressing tumours. We identified ‘HALLMARK_IL6_JAK_STAT3_SIGNALING’ as one of the clearly upregulated pathways (Fig. 6F). This pathway was enriched in FMUUH_RNA-Seq 1 and FMUUH_RNA-Seq 2 (Fig. S14E-F). These above results suggested that the IL6/JAK2/STAT3 signaling may be a downstream pathway of the CircUBA2/miR-144-5p/STC1 axis.

The IL6/JAK2/STAT3 signaling pathway is believed to be closely associated with GC development [24]. Western blotting showed that the total levels of JAK2 and STAT3 remained unchanged in circUBA2-overexpressing cells, whereas the levels of phosphorylated JAK2, STAT3, IL-6, Il-6R, and glycoprotein 130 (gp130) were significantly increased. The opposite result was observed in sh-circUBA2 cells (Fig. 6G). Rescue experiments with circUBA2 and STC1 yielded similar results (Fig. 6H). In addition, by treating circUBA2 knockdown or control cells with different doses of IL-6, we found that IL-6 promoted circUBA2-induced IL-6R, gp130, p-JAK2, and p-STAT3 expression (Fig. 6I). The IL-6 pathway is divided into classical IL-6 and trans-signaling pathways. Subcellular fractionation revealed that the levels of sIL-6R and membrane-bound gp130 decreased the most (Fig. 6J). In the co-immunoprecipitation assay, gp130 and IL-6R levels decreased in sh-circUBA2 cells (Fig. 6K). This suggests that circUBA2 knockdown induces fewer gp130 and IL-6R interactions in vivo. Therefore, we suggest that circUBA2/miR-144-5p/STC1 regulates the selective splicing of IL-6R mRNA or the proteolytic cleavage of IL-6R to amplify IL-6 trans-signaling via sIL-6R, but not through the enhancement of classical IL-6 signalling by IL-6R binding to the cell membrane.

In summary, CircUBA2/miR-144-5p/STC1 regulates IL-6 expression and activates JAK2/STAT3 signaling.

### CircUBA2 activates IL-6/JAK2/STAT3 signaling to mediate GC stemness

**Fig. 7:**
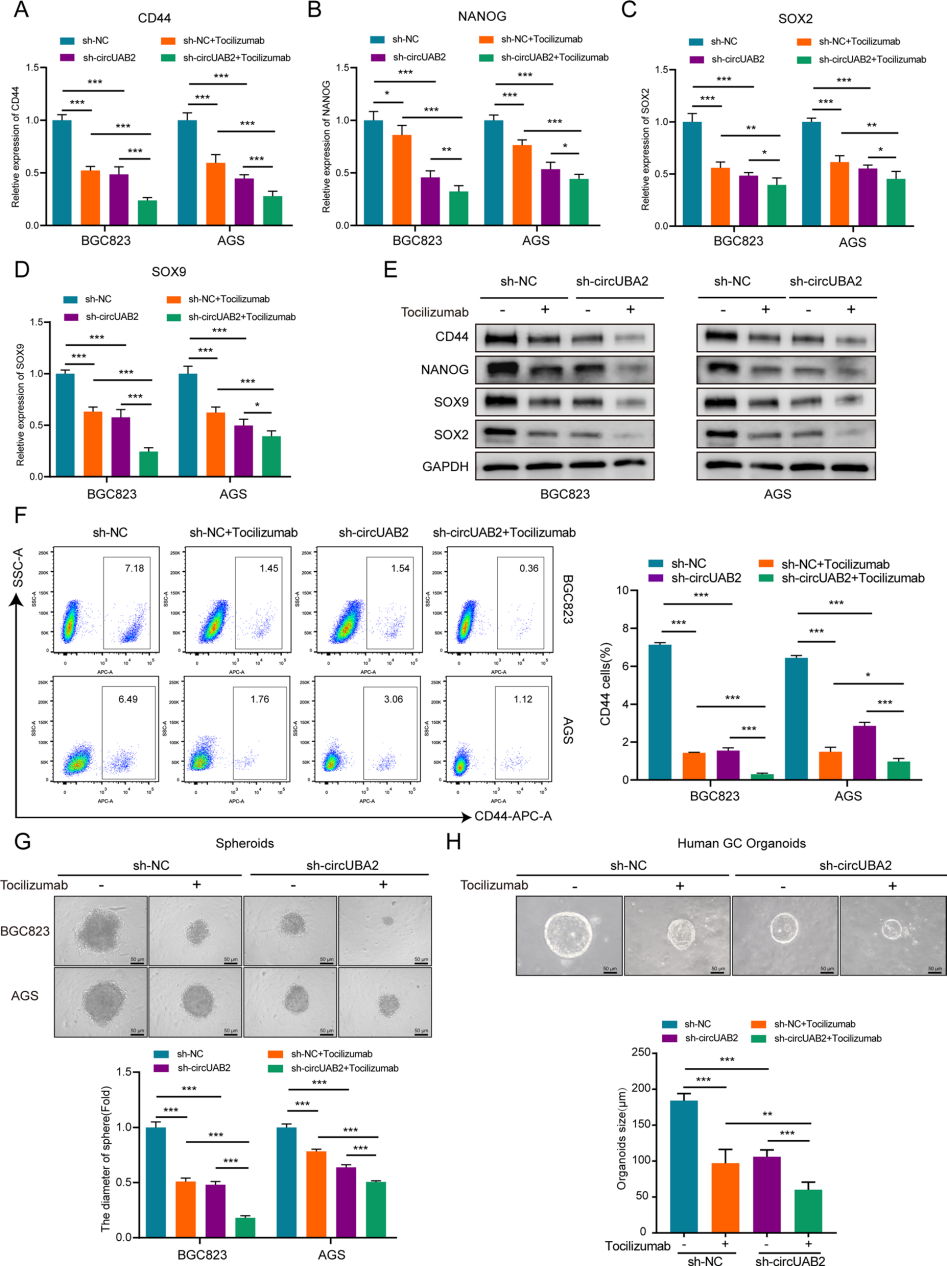
CircUBA2 activates IL-6/JAK2/STAT3 signaling to mediate GC stemness. **A**–**D** The mRNA levels of CD44, NANOG, SOX2, and SOX9 were quantified by qRT-PCR in tocilizumab-treated BGC823 and AGS cells. **E** Protein levels of CD44, NANOG, SOX2, and SOX9 in tocilizumab–treated BGC823 and AGS cells were quantified by western blotting. **F** Proportion of CD44-positive BGC823 and AGS cells and statistical results. **G** Stable transfection of knockdown circUBA2 and control lentivirus in spherical BGC823 and AGS cells treated with tocilizumab, showing that the knockdown of circUBA2 and tocilizumab synergistically inhibited spheroids growth, scale bar = 50 μm. **H** Stable transfection of knockdown circUBA2 and control lentivirus in human organoids further treated with tocilizumab, showing that the knockdown of circUBA2 and tocilizumab synergistically inhibited organoid growth, scale bar = 50 μm. *p < 0.05; **p < 0.01; ***p < 0.001

We performed rescue assays to ascertain the effects of circUBA and IL6 on CSCs proliferation. Tocilizumab (IL6R Ab) is an anti-human interleukin-6 receptor (IL-6R)-neutralising antibody that prevents binding between IL-6 and IL-6R, thus inhibiting both classical and trans signaling [25]. We added tocilizumab to cells transfected with sh-circUBA2 and control cells and observed that tocilizumab treatment further suppressed the mRNA and protein levels of CD44, NANOG, SOX2, and SOX9 in the sh-circUBA2 cell line (Fig. 7A–E). Similarly, tocilizumab further reduced the number of CD44-positive sh-circUBA2 cells (Fig. 7F). Moreover, we tested this hypothesis in BGC823 and AGS cells and human GC organoids and found that sh-circUBA2 and tocilizumab synergistically inhibited the growth of GC 3D cell spheroids and human GC organoids (Fig. 7G, H). Consequently, circUBA2 activates IL-6/JAK2/STAT3 signaling to mediate GC stemness.

## Discussion

The circRNA expression spectrum is required to identify novel tumour oncogenic or suppressor circRNAs and reveal their functions and mechanisms [26–30]. In our study, based on circRNA microarray analysis and GC tissues, we found that circUBA2 is notably expressed in GC and is clearly correlated with poor patient prognosis. Functional experiments confirmed that circUBA2 promotes CSC-like properties of GC in 2D cells, 3D spheroid models, organoid models, and mouse models. We also found that circUBA2 could upregulate STC1 expression via miR-144-5p sponging and further activates the IL-6/JAK2/STAT3 signaling pathway, thereby promoting stemness and tumour progression in GC in vivo and in vitro. More importantly, we found that the combination of knockdown circUBA2 and tocilizumab treatment significantly inhibited the stem cell-like properties of GC cells, which may provide a potential therapeutic strategy for refractory GC.

The classical mechanisms underlying circRNA regulation in cancer involve ceRNAs. Specifically, circRNAs competitively bind to miRNAs through miRNA response elements (MRE) and reduce the repressive effect of miRNAs on downstream genes, hence influencing the expression and function of genes [31, 32]. Bioinformatic predictions and experiments indicated that circUBA2 targets miR-144-5p. Furthermore, miR-144-5p is downregulated in multiple cancers, including renal cell carcinoma, non-small cell lung cancer, and GC, and is involved in the regulation of cancer cell proliferation and migration, stem cell differentiation, inflammation, and apoptosis [33–36]. For example, miR-144-5p acts as a tumour suppressor gene and enhances the radiosensitivity of non-small cell lung cancer cells by targeting ATF2 [36]. In GC, miR-144-5p regulates tyrosine 3-monooxygenase/tryptophan 5-monooxygenase activation protein β axis to inhibit cancer cell proliferation and metastasis [35]. Next, STC1 was identified as a downstream target gene of miR-144-5p using bioinformatics and experimental validation, demonstrating the formation of the circUBA2/miR-144-5p/STC1 axis. Therefore, we hypothesised that circUBA2 affects the CSC-like properties of GC and thus tumour progression through upregulation of STC1 expression.

Stanniocalin-1 (STC1), originally discovered in the Stannius corpus of bony fish, is a secreted homodimeric glycoprotein that regulates calcium and phosphate homeostasis [37]. Recent studies have confirmed that STC1 is overexpressed and regulates CSC-like properties in various cancers [38–40]. STC1 is highly expressed in glioma stem cell-like cells. STC1 enhances the stemness characteristics of glioblastoma cells by activating the NOTCH1-SOX2 signaling pathway [39]. Our study showed that STC1 was highly expressed in both the TCGA dataset and GC tissues and was associated with a poor prognosis. In terms of cellular function, we demonstrated that circUBA2 promoted GC stemness by upregulating STC1 expression, thereby promoting the proliferation, migration, and metastasis of GC cells. These results suggested that the circUBA2/miR-144-5p/STC1 axis plays an vital role in the regulation of cancer stem cells.

Using cytokine array analysis, we further investigated the possible downstream effects of STC1 and found that the secretion of IL-6 was reduced after circUBA2 knockdown. GSEA revealed that high STC1 expression was closely related to the enrichment of IL-6/JAK/STAT3 signaling. IL-6 is a cytokine with multiple functions that mediate the response to infection or injury and participates in tumourigenesis caused by activation of JAK2/STAT3 signaling [41]. IL-6 trans-signaling plays a key role in cancer progression. In briefly, IL-6 binds to sIL-6R before binding to gp130 on the cell surface to activate STAT3 [42]. Our data suggest that STC1 knockdown reduces IL-6 secretion and inhibits the expression of molecules downstream of the IL-6/JAK2/STAT3 signaling pathway, whereas circUBA2 overexpression plays a reversible role. Interestingly, the circUBA2/miR-144-5p/STC1 axis may regulate the selective splicing of IL-6R mRNA or the proteolytic cleavage of IL-6R to amplify IL-6 trans-signaling through sIL-6R, instead of enhancing the classical IL-6 signal through membrane-bound IL-6R. Functionally, we demonstrated that the circUBA2/miR-144-5p/STC1 axis promoted GC stemness by activating the IL-6/JAK2/STAT3 signaling pathway.

Targeting the IL-6/JAK2/STAT3 signaling axis may enhance the effectiveness of cancer therapies [43]. Clinical studies have suggested that neutralising IL-6 or suppressing downstream JAK2/STAT3 signaling may be associated with powerful anticancer activity [44]. Tocilizumab is a humanised IL-6R monoclonal antibody (mAb) that inhibits IL-6 signaling [45]. Our study showed that the combination of tocilizumab and circUBA2 knockdown significantly inhibited the CSC-like properties of GC cells and human organs, thus offering a new strategy for the treatment of GC by ameliorating CSC-like properties. Nevertheless, additional clinical studies and theoretical knowledge are required to verify whether treatments targeting circUBA2 using tocilizumab can be adapted in clinical practice.

## Conclusion

In conclusion, we identified an important mechanism in GC: circUBA2 acts as a sponge for miR-144-5p to upregulate the expression level of STC1, which further activates the IL-6/JAK2/STAT3 signaling pathway to promote CSC-like properties and tumour progression in GC and is therefore significantly associated with poor prognosis in GC.

### Supplementary Information

Additional file 1: Supplementary materials and methods.

Additional file 2: Supplementary Table 1-3. Table 1 Primers used in this study. Table 2. Reagents and Kits used in this study. Table 3. Equipment used in this study.

Additional file 3. Figure S1: CircUBA2 expression is upregulated in GC and promotes GC development. (A) Quantification of expression levels of 15 circRNAs in 60 pairs of GC tumour tissues and matched normal tissues by qRT-PCR. (B) BGC823 and AGS cells with stable circUBA2 overexpression and circUBA2 knockdown were created. The changes in circUBA2 and UBA2 expression were confirmed by qRT-PCR. (C) Western blotting of cyclin proteins related to G1/S transition, including cyclin D1, cyclin E1, and CDK2 after circUBA2 alteration in BGC823 and AGS cells. (D) Effects of circUBA2 alteration on cell cycle distribution of BGC823 and AGS cells detected by flow cytometry. (E) The images of xenograft tumours of sacrificed mice subcutaneously injected with indicated cells 22 days after injections (n=5 mice per group). (F) Growth curves of xenograft tumours. (G) The weight of xenograft tumours. *p <0.05; **p <0.01; ***p <0.001.

Additional file 4. Figure S2: CircUBA2 promotes CSC-like properties of GC cells. (A) Several stemness-related factors including CD44, NANOG, SOX2, SOX9, CD24, LGR5, OCT4 and CD133 were measured by qRT-PCR. Colours represent the intensity scale of expression in circUBA2 vs. vector cells and sh-circUBA2 vs. control cells calculated by log2 transformation. (B) Western blotting of CD44, NANOG, SOX9 and SOX2 after circUBA2 alternation in BGC823 and AGS cells. (C) Representative flow cytometric scatter charts and quantification of the CD44 positive proportion of AGS cells. (D) Representative images from the sphere formation assay of AGS cells. Sphere formation abilities were accessed by the number of spheres, scale bar = 200 μm. (E) Human GC organoids with stable circUBA2 overexpression and circUBA2 knockdown were created. The changes in circUBA2 expression were confirmed by qRT-PCR. (F-H) BGC823 cells with/without circUBA2 knockdown were serially diluted and planted into the U-bottom 96-well plates, scale bar = 50 μm. (I-K) BGC cells with/without circUBA2 knockdown were serially diluted and xenografted into nude mice subcutaneously and showed the cell numbers injected and frequency of tumour formation at day 42 (n=5 mice per group). Displays the probability estimates calculated with Extreme Limiting Dilution Analysis (ELDA) software (http://bioinf.wehi.edu.au/software/elda/). A significant difference in tumour formation capacity was observed between the control and sh-circUBA2 groups. *p <0.05; **p <0.01; ***p <0.001.

Additional file 5. Figure S3: CircUBA2 enhances the expression of CD44-NANOG-positive cells. (A-B) IF staining indicating CD44 (red) and NANOG (green) together with DAPI (blue) in BGC823 cells. The percentages of CD44 and NANOG staining results were quantified, scale bar = 50μm. (C-D) IF staining indicating CD44 (red) and NANOG (green) together with DAPI (blue) in AGS cells. The percentages of CD44 and NANOG staining results were quantified, scale bar = 50 μm. *p <0.05; **p <0.01; ***p <0.001.

Additional file 6. Figure S4: The expression of CD44, NANOG, SOX2, and SOX9 in xenograft tumours was detected by IHC and western blotting. (A) CD44,NANOG,SOX2, and SOX9 IHC assays were adapted to detect the sections of nude mouse xenograft tumours injected with the indicated cells, scale bar = 25 μm. (B-E) The results of CD44, NANOG, SOX9 and SOX2 IHC were quantified. (F) Western blotting for CD44, NANOG, SOX9,and SOX2 for xenograft tumours with/without circUBA2 knockdown. *p <0.05; **p <0.01; ***p <0.001.

Additional file 7. Figure S5: The mRNA expression and correlation analysis of miR-181a-5p, miR-181b-5p, and miR-4766-3p in 90 paired GC tissues. (A-C) The mRNA expression of miR-181a-5p, miR-181b-5p, and miR-4766-3p in 90 paired GC and adjacent tissues was determined by qRT-PCR. (D-F) Correlation between circUBA2 and miR-181a-5p, miR-181b-5p or miR-4766-3p according to our GC samples. *p <0.05; **p <0.01; ***p <0.001.

Additional file 8. Figure S6: The colony formation assay and cell migration assay related to circUBA2 and miR-144-5p. (A) BGC823 and AGS cells transfected with a miR-144-5p mimic, or negative control were further transfected with vector or circUBA2 overexpression lentivirus for colony formation assay. (B) BGC823 and AGS cells transfected with a miR-144-5p inhibitor or negative control were further transfected with a control or circUBA2 knockdown lentivirus for colony formation assay. (C) BGC823 and AGS cells transfected with a miR-144-5p mimic, or negative control were further transfected with a vector or circUBA2 overexpression lentivirus for cell migration assay, scale bar = 200 μm. (D) BGC823 and AGS cells transfected with a miR-144-5p inhibitor or negative control were further transfected with a control or circUBA2 knockdown lentivirus for cell migration assay, scale bar = 200 μm. *p <0.05; **p <0.01; ***p <0.001.

Additional file 9. Figure S7: The cell cycle assay related to circUBA2 and miR-144-5p. (A-B) Representative images of cell cycle distribution among indicated cells detected by flow cytometry. (C-D) Western blotting of cyclin proteins related to G1/S transition, including cyclin D1, cyclin E1, and CDK2 in transfected cells. *p <0.05; **p <0.01; ***p <0.001.

Additional file 10. Figure S8: The western blotting, sphere formation assay and flow cytometry related to circUBA2 and miR-144-5p. (A-B) The protein levels of CD44, NANOG, SOX9, SOX2 after circUBA2 and miR-144-5p alternation in transfected BGC823 and AGS cells was detected by western blotting. (C-D) Representative images and quantification of formatted spheres among indicated cells, scale bar = 200 μm. (E-F) Representative flow cytometric scatter charts and quantification of the CD44 positive proportion in BGC823 and AGS transfected with indicated vectors. *p <0.05; **p <0.01; ***p <0.001.

Additional file 11. Figure S9: STC1 expression is upregulated in GC and correlates with poor prognosis. (A-E) Determination of mRNA expression levels of HOXA13, COL8A1, BCAT1, PDSS1, MTPAP in miR-144-5p mimic-treated or inhibitor-treated and control BGC823 and AGS cells by qRT-PCR. (F) Representative images of STC1 protein levels in gastric tumour and adjacent normal tissues. (G) The T/N ratios of the total results described in Fig. S9F. (H) Scoring criteria for STC1 IHC staining results in gastric tissue microarray (TMA). Magnification: x4 and x40, scale bar = 50 µm. (I) Expression of STC1 in 210 paraffin-embedded specimens from the internal cohort was determined by TMA-based IHC staining, scale bar = 50 μm. (J) STC1 IHC score of gastric tumours and adjacent normal tissues in Fig. S9I. Data were presented asthe mean ± SD and were analysed using Student’s t-test. (K) Kaplan-Meier analysis of the correlations between STC1 expression and overall survival in the TMA (p < 0.05). (L-M) Difference in STC1 protein expression between gastric tumours and adjacent normal gastric tissues in the TCGA database. (N) Kaplan-Meier analysis of the correlations between STC1 expression and overall survival in the TCGA database (p < 0.05). *p <0.05; **p <0.01; ***p <0.001.

Additional file 12. Figure S10: The rescue experiment associated with miR-144-5p and STC1 in BGC823 and AGS cells. (A) Representative images and quantification of clone formation. (B) Representative images and quantification of migrated cells among indicated cells tested by transwell assay, scale bar = 200 μm. (C) Representative images of cell cycle distribution among indicated cells detected by flow cytometry. *p <0.05; **p <0.01; ***p <0.001.

Additional file 13. Figure S11: STC1 expression is upregulated in GC and correlates with poor prognosis. (A) Representative images and quantification of clone formation. (B) Representative images and quantification of migrated cells among indicated cells tested by transwell assay, scale bar = 200 μm. (C) Representative images of cell cycle distribution among indicated cells detected by flow cytometry. (D) Western blotting of cyclin proteins related to G1/S transition, including cyclin D1, cyclin E1, and CDK2 in transfected cells. *p <0.05; **p <0.01; ***p <0.001.

Additional file 14. Figure S12: The expression of CD44, NANOG, SOX2, and SOX9 in xenograft tumours was detected by IHC. (A) STC1, CD44, NANOG, SOX9, and SOX2 IHC assays were adapted to detect the sections of nude mouse xenograft tumours injected with the indicated cells , scale bar = 25 μm. (B-F) The results of STC1, CD44, NANOG, SOX9 and SOX2 IHC were quantified. *p <0.05; **p <0.01; ***p <0.001.

Additional file 15. Figure S13: The rescue experiment associated with circUBA2 and STC1 in mice. (A) Representative images of HE stained lung tissues (n=5 mice per group), scale bar = 50 μm. (B) Quantification of lung metastases in mice injected with the indicated cells. (C) Representative images of HE stained liver tissue (n=5 mice per group), scale bar = 50 μm. (D) Quantification of liver metastases in mice injected with the indicated cells. *p <0.05; **p <0.01; ***p <0.001.

Additional file 16. Figure S14: The expression of IL-6R, sIL-6R in GC cells and the GSEA of STC1-related pathway in our centre. (A-B) IL-6R expression in stably transfected BGC823 and AGS cells was determined by qRT-PCR. (C-D) sIL-6R expression in the supernatant of BGC823 and AGS cell was determined by ELISA. (E) GSEA of FMUUH_RNA-Seq 1 revealed a notable relationship between STC1 and ‘HALLMARK_IL6_JAK_STAT3_SIGNALING’ signaling pathway (n=60, p = 0.01131). (F) GSEA of FMUUH_RNA-Seq 2 revealed a notable relationship between STC1 and ‘HALLMARK_IL6_JAK_STAT3_SIGNALING’ signaling pathway (n=60, p = 0.001865). *p <0.05; **p <0.01; ***p <0.001.

## Data Availability

The datasets used and analysed during the current study are available from the corresponding author on reasonable request.

## Abbreviations

CircRNAs: Circular RNAs
GC: Gastric cancer
CSCs: Cancer stem cells
MiRNAs: MicroRNAs
STC1: Stanniocalin-1
TMA: Tissue microarray
FISH: Fluorescence in situ hybridization
IF: Immunofluorescence
RIP: RNA Immunoprecipitation
IL-6: Interleukin-6
gp130: Glycoprotein 130
sIL-6R: Soluble IL-6R
IL-6R: Interleukin-6 receptor
