# Supplementary material for: CircUBA2 promotes the cancer stem cell-like properties of gastric cancer through upregulating STC1 via sponging miR-144-5p
Source: Cancer Cell Int. 2024 Aug 5;24:276. doi: 10.1186/s12935-024-03423-0 (PMC11302268; doi:10.1186/s12935-024-03423-0)
Supplement: Supplementary file 1 — Additional file 1: Supplementary materials and methods. [file 12935_2024_3423_MOESM1_ESM.doc]

**Supplementary** **Materials and Methods**

**Tissue microarray (TMA)**

The TMA contained many GC samples. In brief, the pathologist reviewed all GC tissues and marked representative areas in the paraffin blocks that were free of necrotic and hemorrhagic material. GC samples were fixed onto paraffin blocks using a specific TMA instrument, and HE (hematoxylin and eosin) staining was used to identify the 4-6 μm thick sections that were cut to ensure successful sample preparation.

**Nucleic acid electrophoresis assay**

First, both the specific gDNA and cDNA of circUBA2 as well as UBA2 were extracted from BGC823 cells and then further studied using TAE running buffer and 2% agarose gel electrophoresis. The experimental procedure was to isolate relevant DNA via electrophoresis at 100V for 30 minutes. The assay marker used was the DL1,000 DNA marker (100-1000 bp) (Takara, Japan). The final bands were examined using UV light irradiation (Thermo Fisher Scientific, USA).

**Subcellular Fraction**

The extraction of RNA was carried out on ice, according to the requirements of the Cytoplasmic and Nuclear RNA Purification Kit (NORGEN, Canada). Firstly, lysis of BGC and AGS cells in EP tubes with Lysis Buffer J on a shaker for 10 minutes. The sediment and supernatant, also known as the nuclear and cytoplasmic fractions, were collected after centrifuged for 3 minutes at 14,000 x g. These corresponding RNA was extracted using Buffer SK and washed repeatedly. Finally, qRT-PCR was carried out to check RNA expression in the nucleus or cytoplasm of the cells.

**RNA extraction and quantitative real-time polymerase chain reaction (qRT-PCR)**

Total RNA was isolated from the cell line or tissues using TRIzol reagent (Invitrogen, USA)，and cDNA was synthesized using a Reverse Transcription Reagent Kit (Takara,Japan) according to the manufacturer’s instructions. The expression of mRNAs was measured using SYBR Green PCR Master mix (Takara, Japan) on a real-time PCR instrument (Bio-Rad, USA). The levels of miRNA were normalized to that of small nuclear U6, and GAPDH was used as an internal control for the relative expression of circRNAs and mRNAs. The relative expression levels were calculated using the 2−ΔΔCT method, and the primers used are listed in Additional file 1: Supplementary Table S1.

**Western blotting assay**

Samples and cells were collected for western blotting, western blot analysis was performed using the following antibodies: CD44 (ab254530, Abcam, 1:1000), NANOG (14295-1-AP, Proteintech, 1:1000), SOX9 (bs-4177R, Bioss, 1:1000), SOX2 (ab171380, Abcam, 1:1000), STC1 (20621-1-AP, Proteintech, 1:1000), IL-6 (EM30301, HuaBio, 1:1000), IL-6R (66855-1-Ig, Proteintech, 1:1000), gp130 (bs-9396R, Bioss, 1:1000), STAT3 (A1192, Abclonal, 1:1000), phospho-STAT3 (Tyr705) (bs-1658R, Bioss, 1:1000).

**Cell culture and reagents**

The human GC cell line GES-1, BGC-823, AGS, HGC27, N87, SGC7901, MKN45, MGC803 was obtained from the Cell Line Bank, Chinese Academy of Sciences. GES-1, BGC-823, HGC27, N87, SGC7901, MKN45, MGC803 cells were cultured in RPMI-1640 (Gibco, Grand Island, NY , USA) with 10% foetal bovine serum (FBS) (Gibco, Grand Island, NY, USA), and AGS cells were cultured in DMEM/F12 (Gibco, Grand Island, NY, USA) with 10% FBS (Gibco, Grand Island, NY, USA) at 37°C containing 5% CO2. Overexpression and knockdown lentiviruses for circUBA2, control lentivirus, miR-144-5p mimics, miR-144-5p inhibitor were purchased from GeneChem Corporation (Shanghai, China). SiRNA and shRNA of STC1 were purchased from Santa cruz (USA). Transfection was performed according to the manufacturer’s instructions. Puromycin (2 μg/ml, Sigma) was used to select stable clones for at least 1 week. At the indicated time points, the cells were harvested for mRNA analysis and protein analysis as well as for other assays.

**Cell counting Kit-8 and colony formation assays**

For the CCK-8 assay, 1×10^3^ cells in the logarithmic growth phase were seeded in 96-well plates. In brief, 10 μl of CCK-8 in 1640 medium (100 μl) was added to each well, and the cells were incubated for 2 hours at 37°C. Then, the optical density values were measured at 450 nm on a microplate reader (Bio-Rad, Hercules, CA, USA). The cell growth rate was examined at 1, 2, 3, 4 and 5 days after seeding, and statistical results were obtained from three independent experiments. For the colony formation assay, 1x10³ cells were seeded in 6-well plates and cultured for 7-14 days. Media were changed every other day, and colonies were stained with 0.5% crystal violet for 20 minutes. Only colonies containing more than 50 cells were counted. Statistical results were obtained from three independent experiments.

**Flow cytometry**

For the cell cycle assay, A total of 5x10^5^ cells was seeded in 6 cm plates and incubated for 24 hours. Next, cells were harvested and fixed with 70% ethanol at 4°C overnight. After the cells were washed, they were stained with propidium iodide (PI) and then filtered through a 70μm cell strainer immediately prior to flow cytometry, which was carried out on a FACSVerse flow cytometer (BD Biosciences, San Jose, CA). For detection of the CD44 proportion, 1x10^6^ cells were resuspended in PBS containing 2% FBS and incubated with APC-conjugated CD44 (Invitrogen, 17-0441-82) for 20 min. After incubation, the cells were washed and resuspended in 500 μl of PBS and analyzed using flow cytometry.

**Cell migration assay**

Transwell assays were performed using transwell chambers (polycarbonate filters with 8 μm pores, BD Bioscience). A total of 1×10^5^ cells in 200 μl of serum-free medium were seeded in the upper chamber, and 500 μl of culture medium containing 10% FBS was added to the lower chamber. After 12-24 hours of incubation, cells that migrated or invaded to the bottom chamber were stained with 0.5% crystal violet. Cells were counted in three randomly selected fields (magnification, × 200) per well.

***In* *vitro* limiting dilution assay (LDA)**

Special U-bottom 96-well plates were chosen for the experiments. BGC823 or AGS control cells or circUBA2-knockdown cells were diluted into three groups at different cell levels (100, 10, 1 cells/well). Cells were cultured for three weeks and the number of wells containing spheroids was counted. More precise results were obtained by Extreme Limiting Dilution Analysis (ELDA).

**Immunohistochemistry (IHC) and evaluation**

The serial sections of the FFPE sample were 4 μm and mounted on a glass slide for IHC analysis. The sections were deparaffinized with xylene and rehydrated with alcohol. We blocked the endogenous peroxidase by immersing the slices in a 3% H2O2 aqueous solution for 10 min and microwaved them in 0.01 mol/L sodium citrate buffer (pH 6.0) for 10 min for antigen retrieval. The slides were then washed in phosphate-buffered saline (PBS) and then incubated with 10% normal goat serum (Zhongshan Biotechnology Co., Ltd., China) to eliminate nonspecific reactions. Subsequently, the primary antibody was incubated with the antibody overnight at 4°C. The treatment of the negative control is the same, but the primary antibody is omitted. After rinsing three times with PBS, dilute the slide and secondary antibody for 30 min at room temperature, and develop with diaminobenzidine (DAB) solution. Finally, the slides were counter-stained with hematoxylin, dehydrated and fixed with a cover glass and neutral resin.We performed STC1 (20621-1-AP, Proteintech, 1:200), CD44 (ab254530, Abcam, 1:1000), NANOG (14295-1-AP, Proteintech, 1:500), SOX2 (ab171380, Abcam, 1:100) , SOX9 (bs-4177R, Bioss, 1:200) immunohistochemical staining on subcutaneous xenograft tumours in nude mice. And we performed STC1 immunohistochemical staining on the tumour tissue of GC patients. The staining intensity and average percentage of positive cells in five randomly selected regions were evaluated to represent the protein expression level. The scoring criteria are as follows: staining intensity is divided into 0 (negative staining), 1 (weak staining, light yellow), 2 (medium staining, yellow-brown) or 3 (strong staining, brown), and positive staining of tumour cells. The proportion is divided into 0 (≤5% positive cells), 1 (6-25% positive cells), 2 (26-50% positive cells) or 3 (≥51% positive cells). The final expression is calculated by multiplying the staining intensity score by the proportional staining score (total 0 to 9). Patients with final scores of 0, 1, 2 and 3 were classified as low expression group, and patients with scores of 4, 6 and 9 were classified as high expression group. The IHC results were evaluated by two independent gastroenterology pathologists who were blinded to the clinical data prognosis of the patients. Approximately 90% of the scoring results are the same. When the scores of the two independent pathologists diverged, another pathologist checked the results again and chose one of the scores of the first two doctors, or the three pathologists discussed the decision together.

**Enzyme-linked immunosorbent assay (ELISA)**

ELISA was conducted according to the instructions. Concentrations of IL-6 (human), sIL-6R (human) in the culture supernatant of treated cells were measured with the use of a commercially available kit (MLBIO，Shanghai).

**Processing of public datasets and gene set enrichment analysis (GSEA)**

We used publicly available data from TCGA, which was downloaded from the Genomic Data Commons (https://portal.gdc.cancer.gov) on June 15, 2020, and this download included clinical information and mRNA expression data. Furthermore, we incorporated mRNA expression data obtained from 60 gastric tumor tissues collected at Fujian Medical University Union Hospital in 2021, designated as FMUUH_RNA-Seq 1. Additionally, we included mRNA expression data from another set of 60 gastric tumor tissues collected at the same hospital in 2022, labeled as FMUUH_RNA-Seq 2. The mRNA expression data were presented as counts and were normalized with R software and the “limma” package [1]. Gene set enrichment analysis (GSEA) performed by the Molecular Signature Database (MSigDB) was used to identify the pathways that were significantly enriched in tumour samples [2]. If a gene set had a positive enrichment score, the majority of its members had higher expression accompanied by a higher risk score, and the set was considered ‘enriched’.

**Co-immunoprecipitation (co-IP) assay**

Cells were washed with PBS and lysed in Tris-buffered saline (pH 7.4). Lysates were incubated on ice for 30 minutes before cellular debris and nuclei were removed via centrifugation at 10,000×g for 5 minutes. Cell lysates were incubated with gp130 antibody and IL-6R antibody overnight at 4°C. Protein A-Sepharose (Amersham Biosciences, Piscataway, NJ) beads in a 50:50 mixture in 50 mmol/l Tris buffer, pH 7.0, were added and incubated with the lysates for another 4 hours at 4°C. The immunoprecipitates were washed 4 times in Tris-buffered saline and boiled for 5 minutes in 40 µl of Laemmli buffer containing 0.02% blue bromophenol and 2% beta-mercaptoethanol.

**Immunofluorescence assay**

The organoids on coverslips were rinsed with PBS and fixed with ice-cold 4% paraformaldehyde for 5 minutes at RT. Subsequently, the cells were blocked with 0.2% Triton X-100 for 30 minutes followed by 5% BSA for 1 hour, washed for 30 minutes, and incubated with primary monoclonal antibodies against CD44 and NANOG overnight at 4°C. The next day, the coverslips were incubated for 1 hour in a dark room with fluorescently conjugated secondary antibody (1:200). Furthermore, the coverslips were stained with DAPI (Vector Laboratories, Burlingame, CA, USA) for 5 minutes at 4°C. Finally, a laser scanning confocal microscope (Leica, Germany) was used to observe the expression in cells.

**References**

1. Ritchie ME, Phipson B, Wu D, Hu Y, Law CW, Shi W, et al. limma powers differential expression analyses for RNA-sequencing and microarray studies. Nucleic Acids Res. 2015;43(7):e47.

2. Subramanian A, Tamayo P, Mootha VK, Mukherjee S, Ebert BL, Gillette MA, et al. Gene set enrichment analysis: a knowledge-based approach for interpreting genome-wide expression profiles. Proc Natl Acad Sci U S A. 2005;102(43):15545-50.
