# Supplementary material for: CircUBA2 promotes the cancer stem cell-like properties of gastric cancer through upregulating STC1 via sponging miR-144-5p
Source: Cancer Cell Int. 2024 Aug 5;24:276. doi: 10.1186/s12935-024-03423-0 (PMC11302268; doi:10.1186/s12935-024-03423-0)
Supplement: Supplementary file 2 — Additional file 2: Supplementary Table 1-3. Table 1 Primers used in this study. Table 2. Reagents and Kits used in this study. Table 3. Equipment used in this study. [file 12935_2024_3423_MOESM2_ESM.doc]

**Supplementary Table** 1. Primers used in this study.

| circUBA2 | Forward | AAGGACGTTGAATTTGAAGTTGTT |
| --- | --- | --- |
|  | Reverse | TTCCACATTATAGTCAGGGTTCAT |
| UBA2 mRNA | Forward | AGATCAAAGGCACAGGTTGC |
|  | Reverse | AGCTGCCAGGCACATTCTAT |
| CD44 | Forward | CTGCCGCTTTGCAGGTGTA |
|  | Reverse | CATTGTGGGCAAGGTGCTATT |
| NANOG | Forward | TTTGTGGGCCTGAAGAAAACT |
|  | Reverse | AGGGCTGTCCTGAATAAGCAG |
| SOX2 | Forward | GACAGTTACGCGCACATGAA |
|  | Reverse | TAGGTCTGCGAGCTGGTCAT |
| SOX9 | Forward | GCACTTGCACAACGCCGAG |
|  | Reverse | CCGTTCTTCACCGACTTCCTCC |
| miR-144-5p | Forward | CCGCGCCGGATATCATCATATAC |
|  | Reverse | AGTGCAGGGTCCGAGGTATT |
|  | RT | GTCGTATCCAGTGCAGGGTCCGAGGTATTCGCACTGGATACGACCTTACA |
| miR-181a-5p | Forward | CCGCAACATTCAACGCTGTCG |
|  | Reverse | AGTGCAGGGTCCGAGGTATT |
|  | RT | GTCGTATCCAGTGCAGGGTCCGAGGTATTCGCACTGGATACGACACTCAC |
| miR-181b-5p | Forward | CCGCGAACATTCATTGCTGTCG |
|  | Reverse | AGTGCAGGGTCCGAGGTATT |
|  | RT | GTCGTATCCAGTGCAGGGTCCGAGGTATTCGCACTGGATACGACACCCAC |
| miR-4766-3p | Forward | CCGCGCATAGCAATTGCTCTT |
|  | Reverse | AGTGCAGGGTCCGAGGTATT |
|  | RT | GTCGTATCCAGTGCAGGGTCCGAGGTATTCGCACTGGATACGACACCCAC |
| U6 | Forward | CTCGCTTCGGCAGCACA |
|  | Reverse | AACGCTTCACGAATTTGCGT |
| GAPDH | Forward | CAATGACCCCTTCATTGACC |
|  | Reverse | TTGATTTTGGAGGGATCTCG |
| STC1 | Forward | GTGGCGGCTCAAAACTCAG |
|  | Reverse | GTGGAGCACCTCCGAATGG |
| HOXA13 | Forward | CTGCCCTATGGCTACTTCGG |
|  | Reverse | CCGGCGGTATCCATGTACT |
| COL8A1 | Forward | GCTGCCACCTCAAATTCCTC |
|  | Reverse | CTTTCTTGGGTACGGCTTCCT |
| BCAT1 | Forward | AGCCCTGCTCTTTGTACTCTT |
|  | Reverse | CCAGGCTCTTACATACTTGGGA |
| PDSS1 | Forward | AACATCTGCCTGTCCAAATGTAT |
|  | Reverse | CGAGTTTGAAAGGATCGGTGTAT |
| MAPAP | Forward | GGGCTCTTGACCCGTTTGAA |
|  | Reverse | TCGTCTCTCCTAAGGTCTTTGG |
| IL-6 | Forward | ATGCAATAACCACCCCTGAC |
|  | Reverse | GCGCAGAAATGAGATGAGTTGT |
| IL-6R | Forward | CTCTGAAGGAAGGCAAGACAAG |
|  | Reverse | GAGATGAGAGGAACAAGCACTG |
| hsa_circ_0004036 | Forward | CTCACAAGCAGCCTTTACCT |
|  | Reverse | ACGACTCAACTCTCCTGTATCT |
| hsa_circ_0044234 | Forward | TGGTGGAGTGTTTAATAAGCAGAA |
|  | Reverse | GGTGTAGTACAACTGTGTCCTT |
| hsa_circ_0087631 | Forward | CATGGGTGGATGTGGAGTTC |
|  | Reverse | TCTTCTAGGAGTCCGCTTCTG |
| hsa_circ_0052372 | Forward | CTGTTTTGTGAGAGCTGTGATACT |
|  | Reverse | CAATAGACAGTACGTTCACCATCC |
| hsa_circ_0010027 | Forward | TCAACAGTGTAACAGGCATTCG |
|  | Reverse | GGAGTCACCACATCATCTTCG |
| hsa_circ_0059880 | Forward | ACACATTTCTGGAGCACATCAA |
|  | Reverse | CCAACCTTGTAGACGGCATT |
| hsa_circ_0092303 | Forward | CTCCCTTCCCAAGGTCTGTC |
|  | Reverse | TTCCCACTTAAGCCCCTCAA |
| hsa_circ_0007333 | Forward | AAGCACGATTCCTTCCAACC |
|  | Reverse | TCAGAGTCAATATCAGAGCCTTCA |
| hsa_circ_0006517 | Forward | CCTGGACGCTGAGATTGACT |
|  | Reverse | GAGGTGGTGGAGGAGGAAAG |
| hsa_circ_0001530 | Forward | CTTCTCTGTGTTGTGCTGTCA |
|  | Reverse | ACCAAAGGCTCAAGGAAACC |
| hsa_circ_0018225 | Forward | CTACAGGTGCTTGCCACTATG |
|  | Reverse | CACGCCATTCTTCAGAGTCAT |
| hsa_circ_0006186 | Forward | AGCATAACCAATGGCAGTGAT |
|  | Reverse | GCTAAATGAACTCTCAAAGTCTCG |
| hsa_circ_0050545 | Forward | AAGCATAACCAATGGCAGTGA |
|  | Reverse | TGTCAACAGATACCTGATGTCATC |
| hsa_circ_0050547 | Forward | AAGCATAACCAATGGCAGTGAT |
|  | Reverse | GCATTCGTTTCTTCTCCTTGACT |

**Supplementary Table 2: Reagents and Kits** used in this study.

| **Name** | **Catalogue Number** | **Source** |
| --- | --- | --- |
| Ultra-low attachment 6-well dishes | 3471 | Corning |
| Matrigel | 356255 | Corning |
| B-27 | 17504044 | Life Technologies |
| L-glutamine | 25030149 | Life Technologies |
| Magna RIP™ RNA Binding Protein  Immunoprecipitation Kit | 17-701 | Millipore |
| IL-6 (human), sIL-6R (human)  commercially available kit | ml058097-C/YJ038116 | MLBIO |
| Cytoplasmic and Nuclear  RNA Purification Kit | NGB-21000 | NORGEN |
| Dual-Luciferase Reporter  Assay System | E1910 | Promega |
| RNeasy MinElute Cleanup Kit | 74204 | Qiagen |
| DMSO | D2650 | Sigma |
| Actinomycin D | SBR00013 | Sigma |
| Collagenase A | 10103578001 | Sigma |
| Sucrose | S7903 | Sigma |
| D-sorbitol | S1876 | Sigma |
| Organoid Growth Medium | 6010 | StemCell Technologies |
| DL1,000 DNA marker (100-1000 bp) | 3591A | Takara |
| DAPI | NC9524612 | Vector Laboratories |
| Human Ago2 antibody | SAB4200724 | Millipore |
| Mouse IgG antibody | AP160 | Millipore |
| U-bottom 96-well plates | 7007 | Corning |
| Ultra-low attachment 6-well dishes | 3471 | Corning |
| TRIzol Reagent | 15596026 | Invitrogen |
| Reverse Transcription Reagent Kit | RR047A | Takara |
| SYBR Green PCR Master mix | RR420A | Takara |
| RPMI-1640 | 11875093 | Gibco |
| FBS | 10099-141 | Gibco |
| DMEM/F12 | 11320033 | Gibco |
| Puromycin | 540411 | Sigma |

**Supplementary Table 3: Equipment used in this study.**

| **Name** | **Source** |
| --- | --- |
| E-Gel™ Power Snap | Thermo Fisher Scientific, USA |
| ImageQuant LAS 4000 Mini | General Electric Company, USA |
| IVIS SPECTRUM | PerkinElmer, USA |
| FACSVerse | BD Biosciences, USA |
| Ultra-pure Water System | Millipore, USA |
| Inverted Microscope | Leica, Germany |
| Fluorescent Microscopy | Carl Zeiss, Germany |
| Conventional Centrifuge | Zhongke Zhongjia, China |
| CO2 Incubator | Thermo Fisher Scientific, USA |
| Ultracentrifuge | HITACHI, Japan |
| Ultra-low Temperature Freezer | Eppendorf, Germany |
| Magnetic Stirrer | Da Long, China |
| Tissue Homogenizer | MP Biomedicals, USA |
| Electronic Analytical Balance | METTLER TOLEDO, Switzerland |
| Countstar Bio Tech | Countstar, China |
| Electrophoresis System | Bio-Rad, USA |
| Bechtop | An Tai, China |
| Real-time Fluorescence Quantitative  PCR Instrument | Roche, Switzerland |
| MiniAmp PCR System | Applied Biosystems, USA |
| Micro UV-Vis Spectrophotometer | Thermo Fisher Scientific, USA |
| Microplate Reader | BioTek, USA |
| Low Speed Freezing Centrifuge | Eppendorf, Germany |
| Thermostatic Water Bath | Jing Hong, China |
| Nikon Eclipse CI | Nikon, Japan |
| Laser Scanning Confocal Microscope | Leica, Germany |
