# Supplementary figures and images for: CircUBA2 promotes the cancer stem cell-like properties of gastric cancer through upregulating STC1 via sponging miR-144-5p
Source: Cancer Cell Int. 2024 Aug 5;24:276. doi: 10.1186/s12935-024-03423-0 (PMC11302268; doi:10.1186/s12935-024-03423-0)

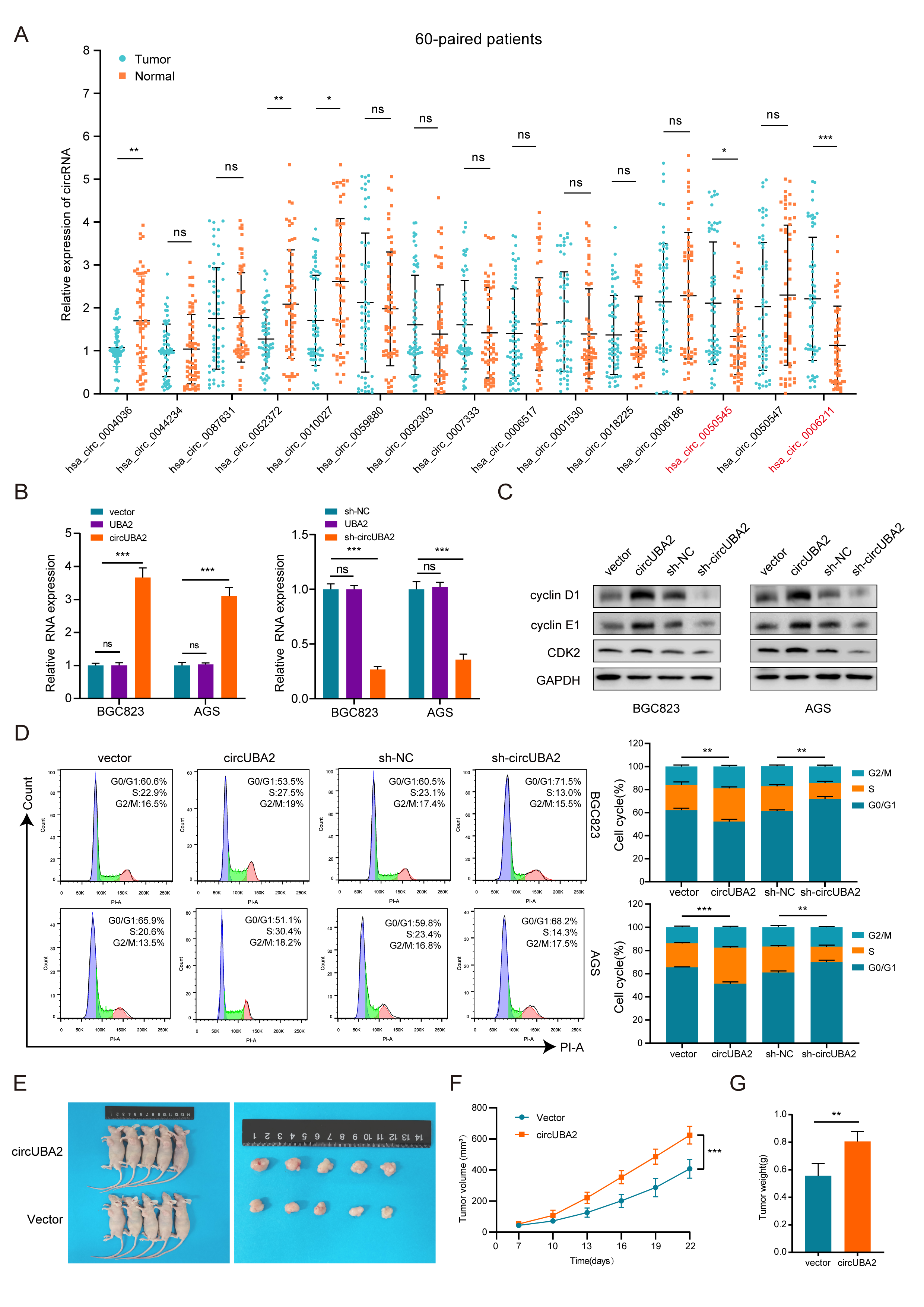

Supplement: Supplementary file 3 — Additional file 3. Figure S1: CircUBA2 expression is upregulated in GC and promotes GC development. (A) Quantification of expression levels of 15 circRNAs in 60 pairs of GC tumour tissues and matched normal tissues by qRT-PCR. (B) BGC823 and AGS cells with stable circUBA2 overexpression and circUBA2 knockdown were created. The changes in circUBA2 and UBA2 expression were confirmed by qRT-PCR. (C) Western blotting of cyclin proteins related to G1/S transition, including cyclin D1, cyclin E1, and CDK2 after circUBA2 alteration in BGC823 and AGS cells. (D) Effects of circUBA2 alteration on cell cycle distribution of BGC823 and AGS cells detected by flow cytometry. (E) The images of xenograft tumours of sacrificed mice subcutaneously injected with indicated cells 22 days after injections (n=5 mice per group). (F) Growth curves of xenograft tumours. (G) The weight of xenograft tumours. *p <0.05; **p <0.01; ***p <0.001. [file 12935_2024_3423_MOESM3_ESM.tif]

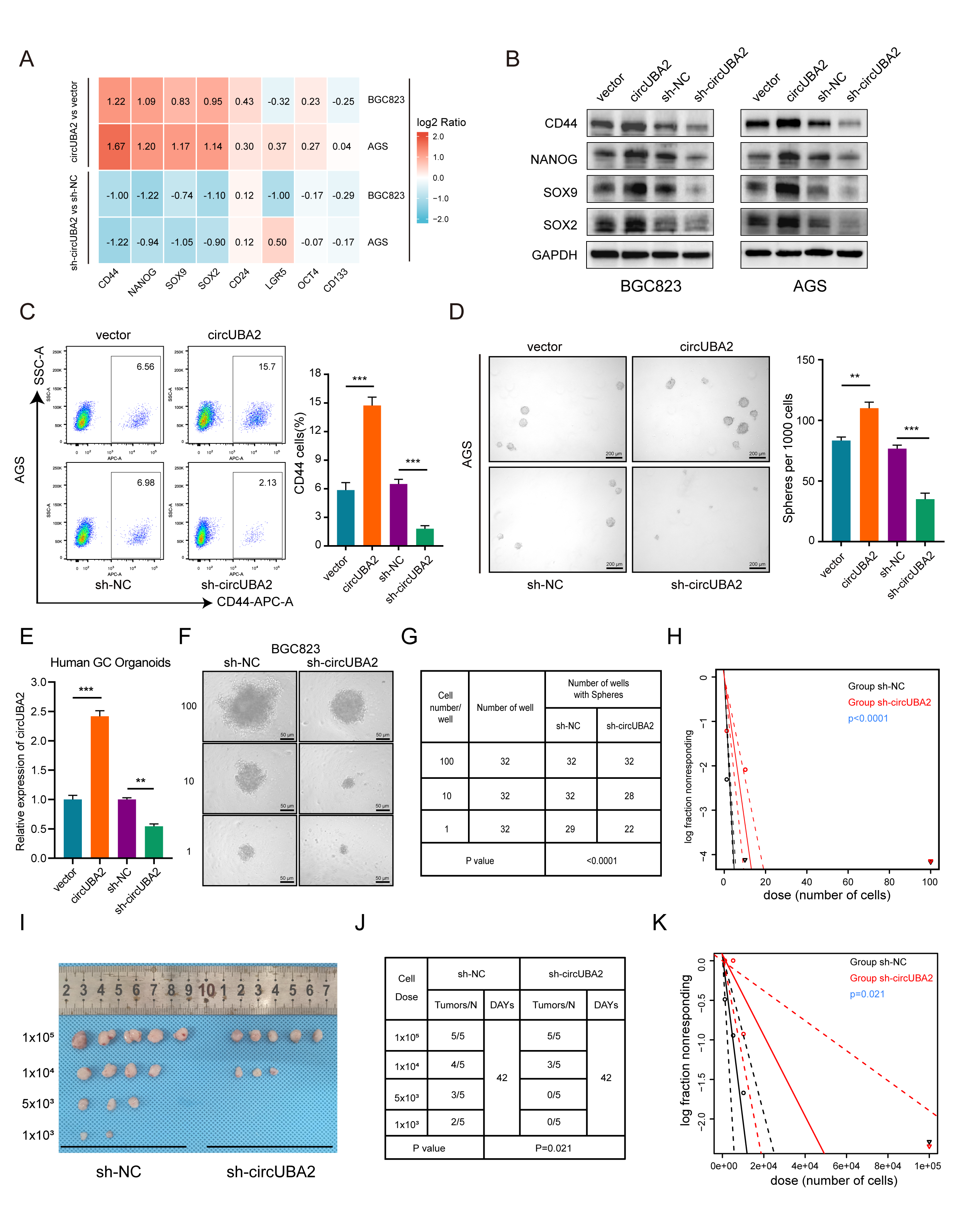

Supplement: Supplementary file 4 — Additional file 4. Figure S2: CircUBA2 promotes CSC-like properties of GC cells. (A) Several stemness-related factors including CD44, NANOG, SOX2, SOX9, CD24, LGR5, OCT4 and CD133 were measured by qRT-PCR. Colours represent the intensity scale of expression in circUBA2 vs. vector cells and sh-circUBA2 vs. control cells calculated by log2 transformation. (B) Western blotting of CD44, NANOG, SOX9 and SOX2 after circUBA2 alternation in BGC823 and AGS cells. (C) Representative flow cytometric scatter charts and quantification of the CD44 positive proportion of AGS cells. (D) Representative images from the sphere formation assay of AGS cells. Sphere formation abilities were accessed by the number of spheres, scale bar = 200 μm. (E) Human GC organoids with stable circUBA2 overexpression and circUBA2 knockdown were created. The changes in circUBA2 expression were confirmed by qRT-PCR. (F-H) BGC823 cells with/without circUBA2 knockdown were serially diluted and planted into the U-bottom 96-well plates, scale bar = 50 μm. (I-K) BGC cells with/without circUBA2 knockdown were serially diluted and xenografted into nude mice subcutaneously and showed the cell numbers injected and frequency of tumour formation at day 42 (n=5 mice per group). Displays the probability estimates calculated with Extreme Limiting Dilution Analysis (ELDA) software (http://bioinf.wehi.edu.au/software/elda/). A significant difference in tumour formation capacity was observed between the control and sh-circUBA2 groups. *p <0.05; **p <0.01; ***p <0.001. [file 12935_2024_3423_MOESM4_ESM.tif]

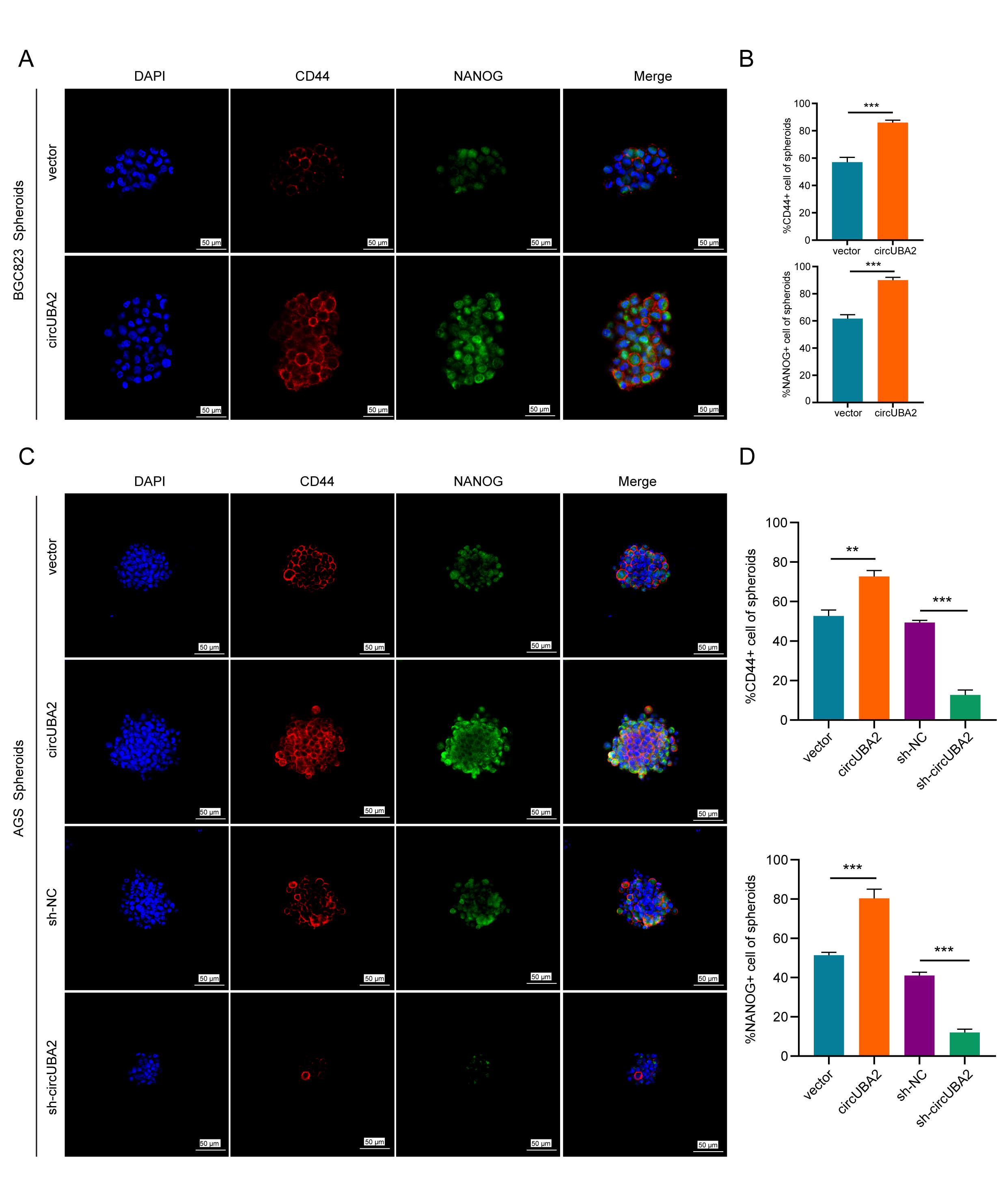

Supplement: Supplementary file 5 — Additional file 5. Figure S3: CircUBA2 enhances the expression of CD44-NANOG-positive cells. (A-B) IF staining indicating CD44 (red) and NANOG (green) together with DAPI (blue) in BGC823 cells. The percentages of CD44 and NANOG staining results were quantified, scale bar = 50μm. (C-D) IF staining indicating CD44 (red) and NANOG (green) together with DAPI (blue) in AGS cells. The percentages of CD44 and NANOG staining results were quantified, scale bar = 50 μm. *p <0.05; **p <0.01; ***p <0.001. [file 12935_2024_3423_MOESM5_ESM.tif]

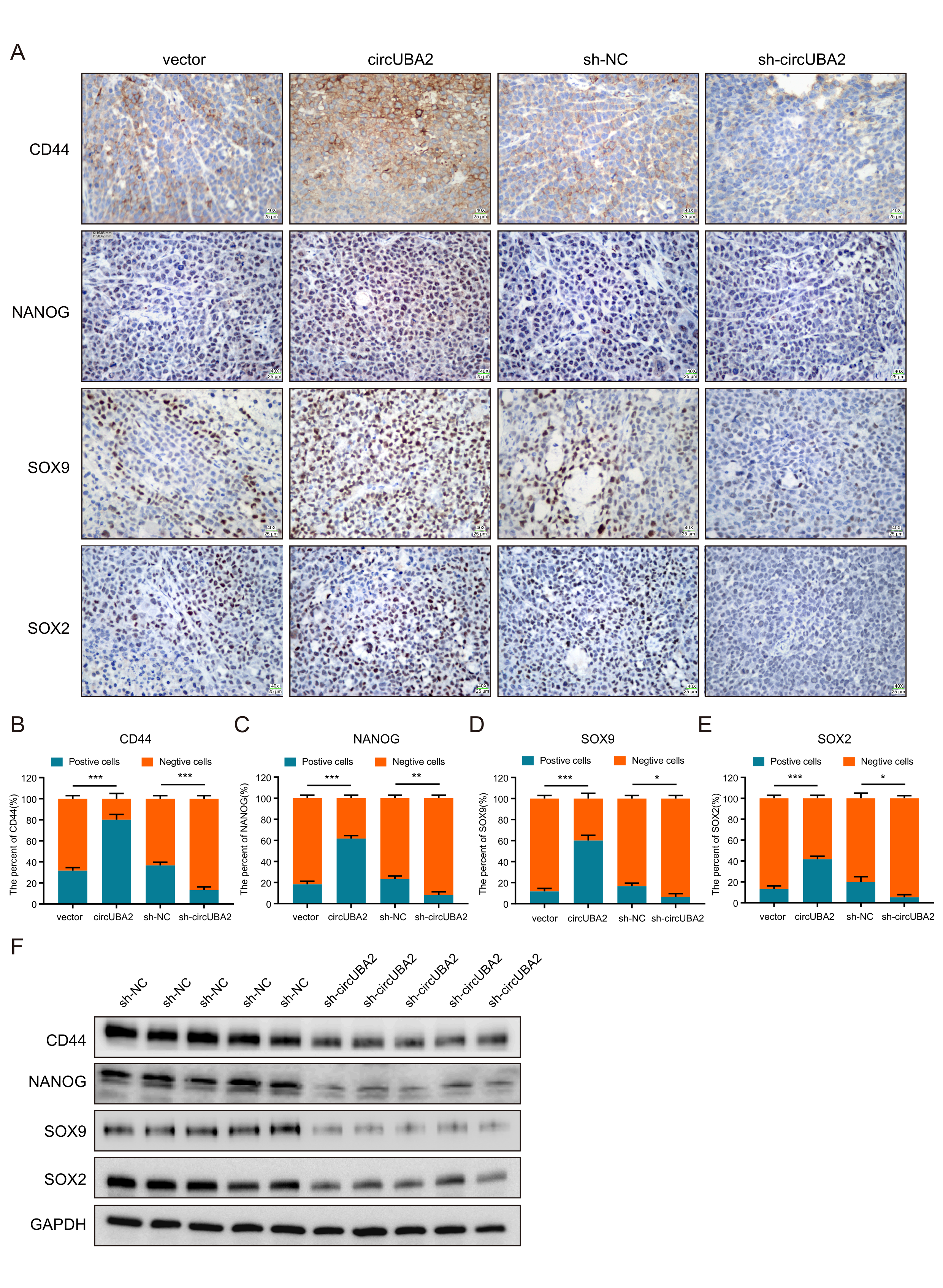

Supplement: Supplementary file 6 — Additional file 6. Figure S4: The expression of CD44, NANOG, SOX2, and SOX9 in xenograft tumours was detected by IHC and western blotting. (A) CD44,NANOG,SOX2, and SOX9 IHC assays were adapted to detect the sections of nude mouse xenograft tumours injected with the indicated cells, scale bar = 25 μm. (B-E) The results of CD44, NANOG, SOX9 and SOX2 IHC were quantified. (F) Western blotting for CD44, NANOG, SOX9,and SOX2 for xenograft tumours with/without circUBA2 knockdown. *p <0.05; **p <0.01; ***p <0.001. [file 12935_2024_3423_MOESM6_ESM.tif]

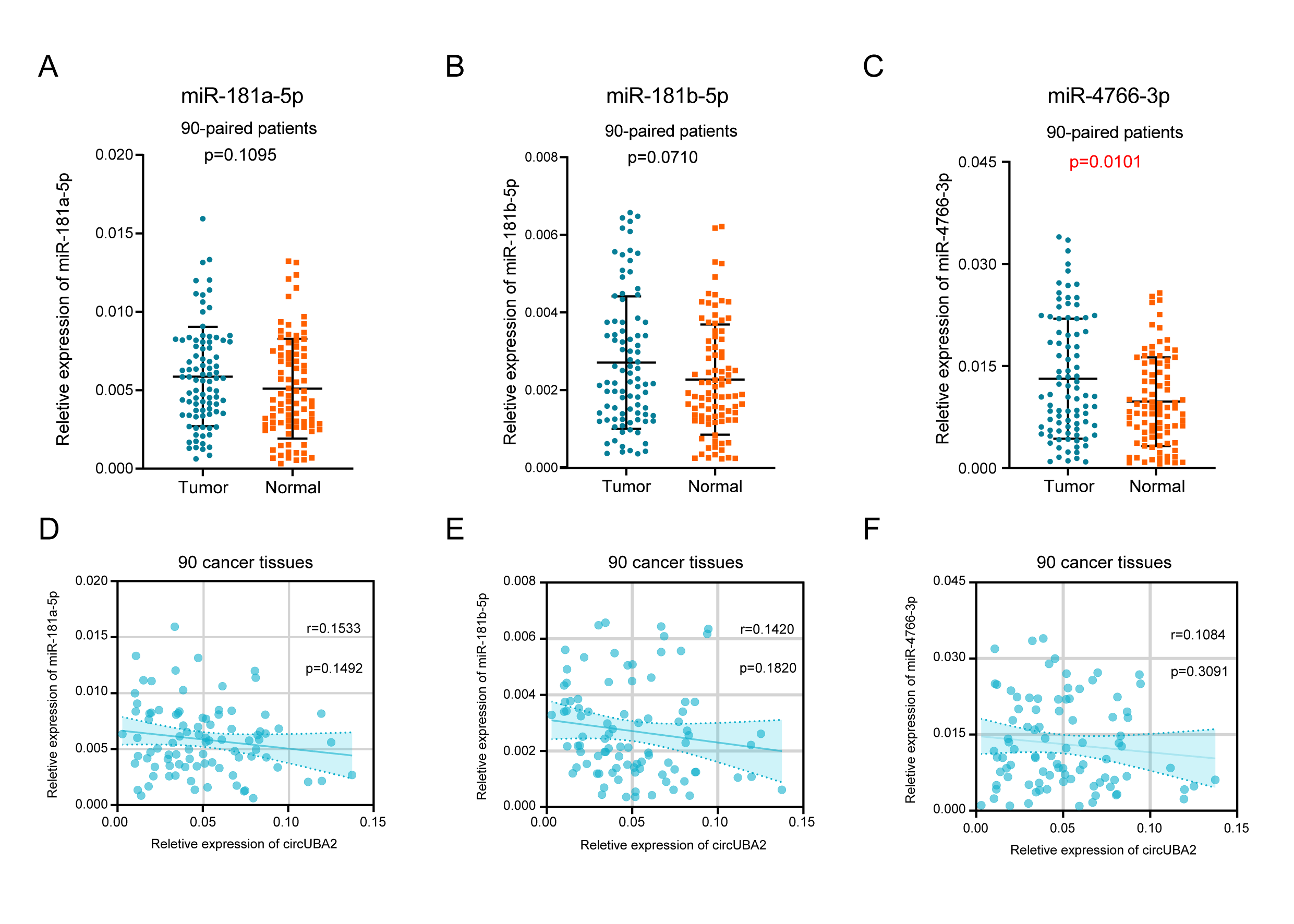

Supplement: Supplementary file 7 — Additional file 7. Figure S5: The mRNA expression and correlation analysis of miR-181a-5p, miR-181b-5p, and miR-4766-3p in 90 paired GC tissues. (A-C) The mRNA expression of miR-181a-5p, miR-181b-5p, and miR-4766-3p in 90 paired GC and adjacent tissues was determined by qRT-PCR. (D-F) Correlation between circUBA2 and miR-181a-5p, miR-181b-5p or miR-4766-3p according to our GC samples. *p <0.05; **p <0.01; ***p <0.001. [file 12935_2024_3423_MOESM7_ESM.tif]

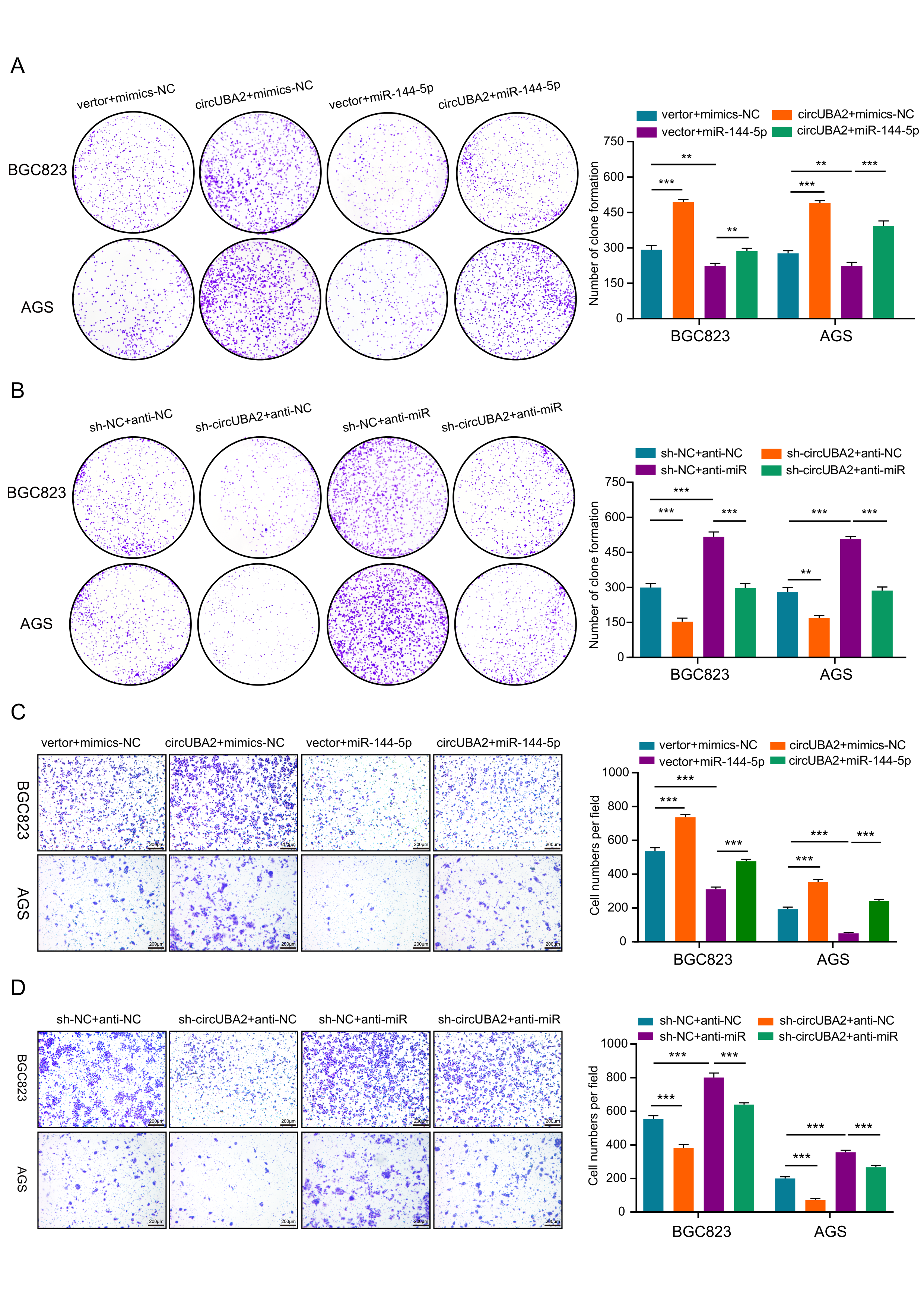

Supplement: Supplementary file 8 — Additional file 8. Figure S6: The colony formation assay and cell migration assay related to circUBA2 and miR-144-5p. (A) BGC823 and AGS cells transfected with a miR-144-5p mimic, or negative control were further transfected with vector or circUBA2 overexpression lentivirus for colony formation assay. (B) BGC823 and AGS cells transfected with a miR-144-5p inhibitor or negative control were further transfected with a control or circUBA2 knockdown lentivirus for colony formation assay. (C) BGC823 and AGS cells transfected with a miR-144-5p mimic, or negative control were further transfected with a vector or circUBA2 overexpression lentivirus for cell migration assay, scale bar = 200 μm. (D) BGC823 and AGS cells transfected with a miR-144-5p inhibitor or negative control were further transfected with a control or circUBA2 knockdown lentivirus for cell migration assay, scale bar = 200 μm. *p <0.05; **p <0.01; ***p <0.001. [file 12935_2024_3423_MOESM8_ESM.tif]

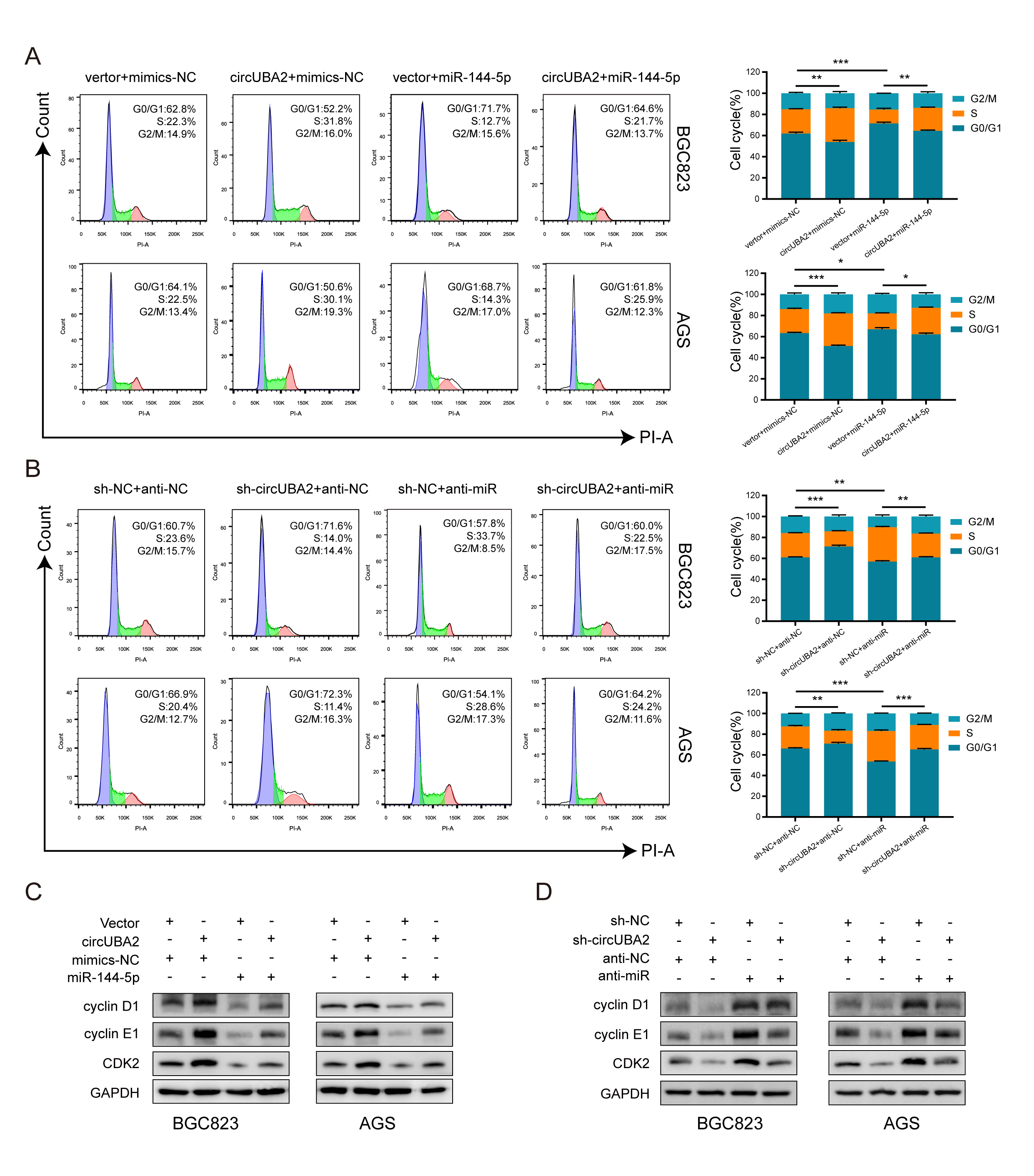

Supplement: Supplementary file 9 — Additional file 9. Figure S7: The cell cycle assay related to circUBA2 and miR-144-5p. (A-B) Representative images of cell cycle distribution among indicated cells detected by flow cytometry. (C-D) Western blotting of cyclin proteins related to G1/S transition, including cyclin D1, cyclin E1, and CDK2 in transfected cells. *p <0.05; **p <0.01; ***p <0.001. [file 12935_2024_3423_MOESM9_ESM.tif]

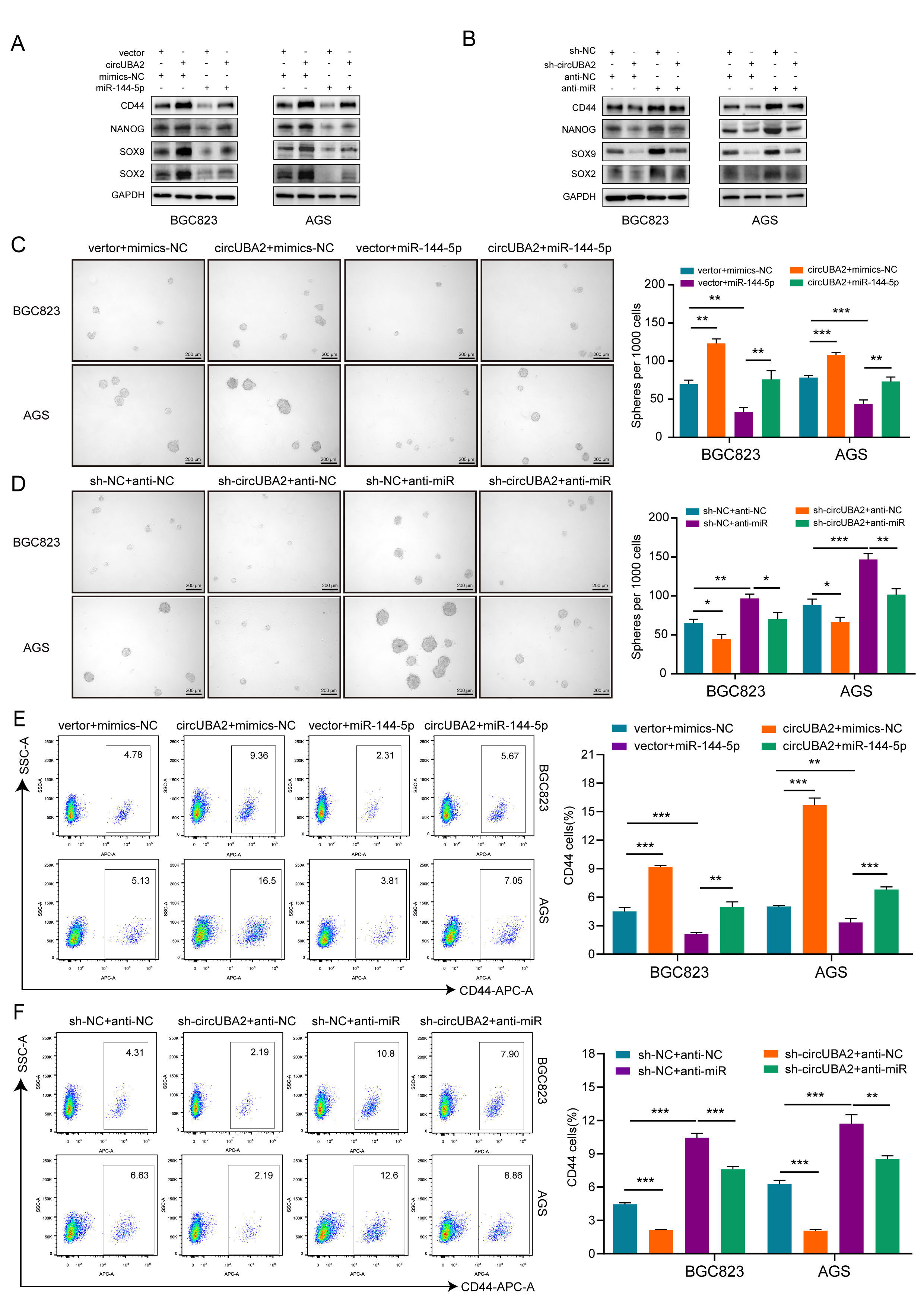

Supplement: Supplementary file 10 — Additional file 10. Figure S8: The western blotting, sphere formation assay and flow cytometry related to circUBA2 and miR-144-5p. (A-B) The protein levels of CD44, NANOG, SOX9, SOX2 after circUBA2 and miR-144-5p alternation in transfected BGC823 and AGS cells was detected by western blotting. (C-D) Representative images and quantification of formatted spheres among indicated cells, scale bar = 200 μm. (E-F) Representative flow cytometric scatter charts and quantification of the CD44 positive proportion in BGC823 and AGS transfected with indicated vectors. *p <0.05; **p <0.01; ***p <0.001. [file 12935_2024_3423_MOESM10_ESM.tif]

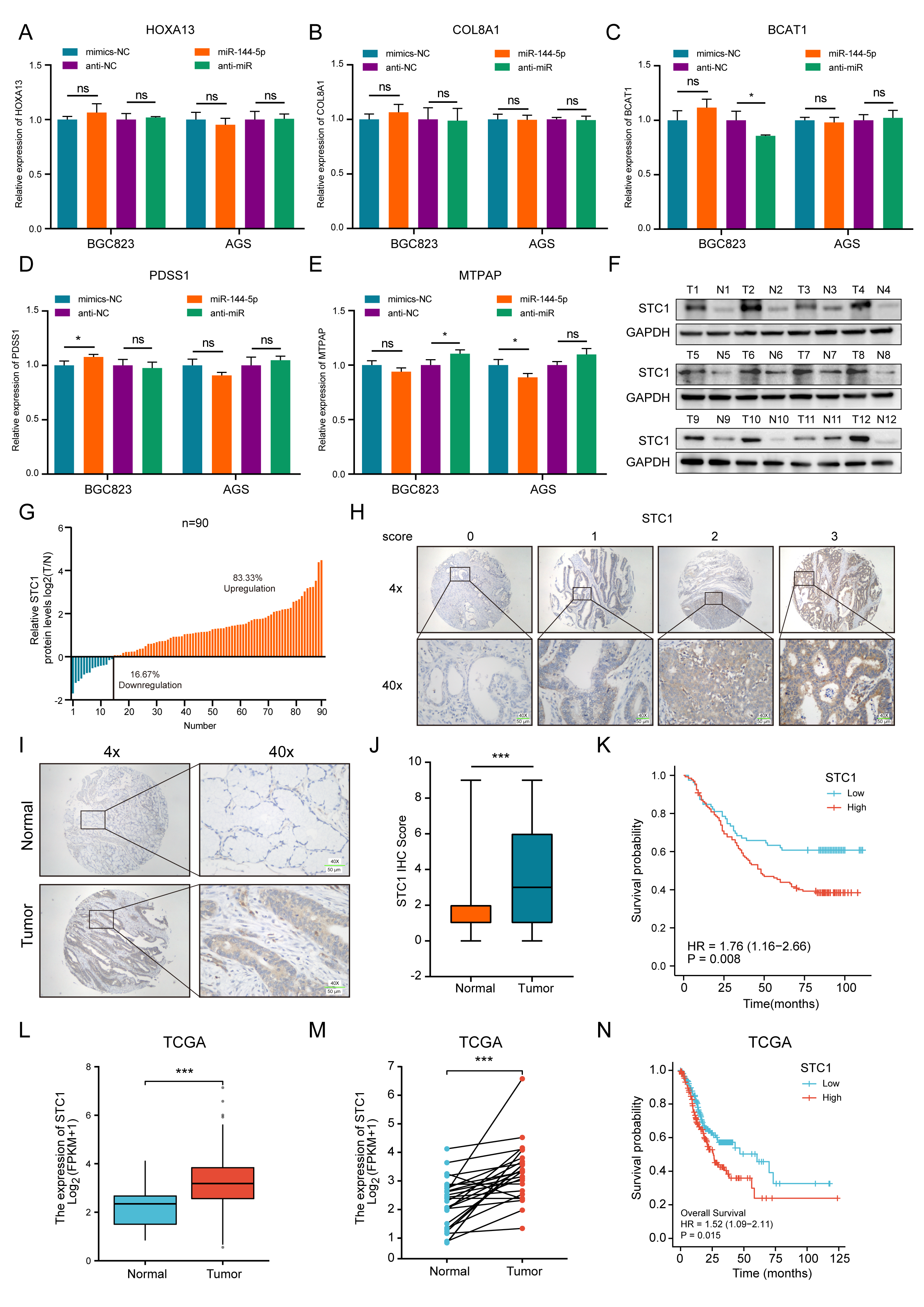

Supplement: Supplementary file 11 — Additional file 11. Figure S9: STC1 expression is upregulated in GC and correlates with poor prognosis. (A-E) Determination of mRNA expression levels of HOXA13, COL8A1, BCAT1, PDSS1, MTPAP in miR-144-5p mimic-treated or inhibitor-treated and control BGC823 and AGS cells by qRT-PCR. (F) Representative images of STC1 protein levels in gastric tumour and adjacent normal tissues. (G) The T/N ratios of the total results described in Fig. S9F. (H) Scoring criteria for STC1 IHC staining results in gastric tissue microarray (TMA). Magnification: x4 and x40, scale bar = 50 µm. (I) Expression of STC1 in 210 paraffin-embedded specimens from the internal cohort was determined by TMA-based IHC staining, scale bar = 50 μm. (J) STC1 IHC score of gastric tumours and adjacent normal tissues in Fig. S9I. Data were presented asthe mean ± SD and were analysed using Student’s t-test. (K) Kaplan-Meier analysis of the correlations between STC1 expression and overall survival in the TMA (p < 0.05). (L-M) Difference in STC1 protein expression between gastric tumours and adjacent normal gastric tissues in the TCGA database. (N) Kaplan-Meier analysis of the correlations between STC1 expression and overall survival in the TCGA database (p < 0.05). *p <0.05; **p <0.01; ***p <0.001. [file 12935_2024_3423_MOESM11_ESM.tif]

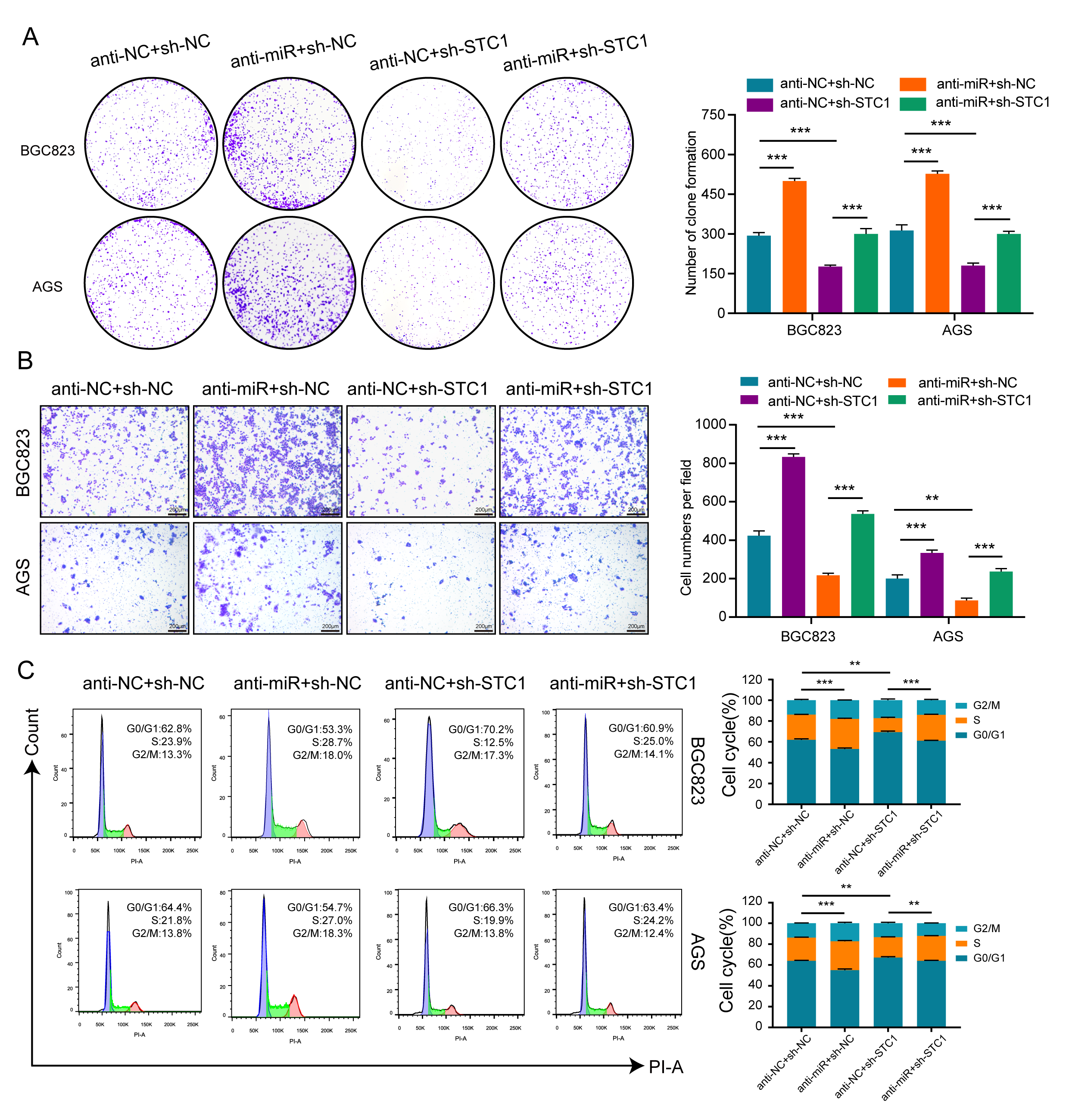

Supplement: Supplementary file 12 — Additional file 12. Figure S10: The rescue experiment associated with miR-144-5p and STC1 in BGC823 and AGS cells. (A) Representative images and quantification of clone formation. (B) Representative images and quantification of migrated cells among indicated cells tested by transwell assay, scale bar = 200 μm. (C) Representative images of cell cycle distribution among indicated cells detected by flow cytometry. *p <0.05; **p <0.01; ***p <0.001. [file 12935_2024_3423_MOESM12_ESM.tif]

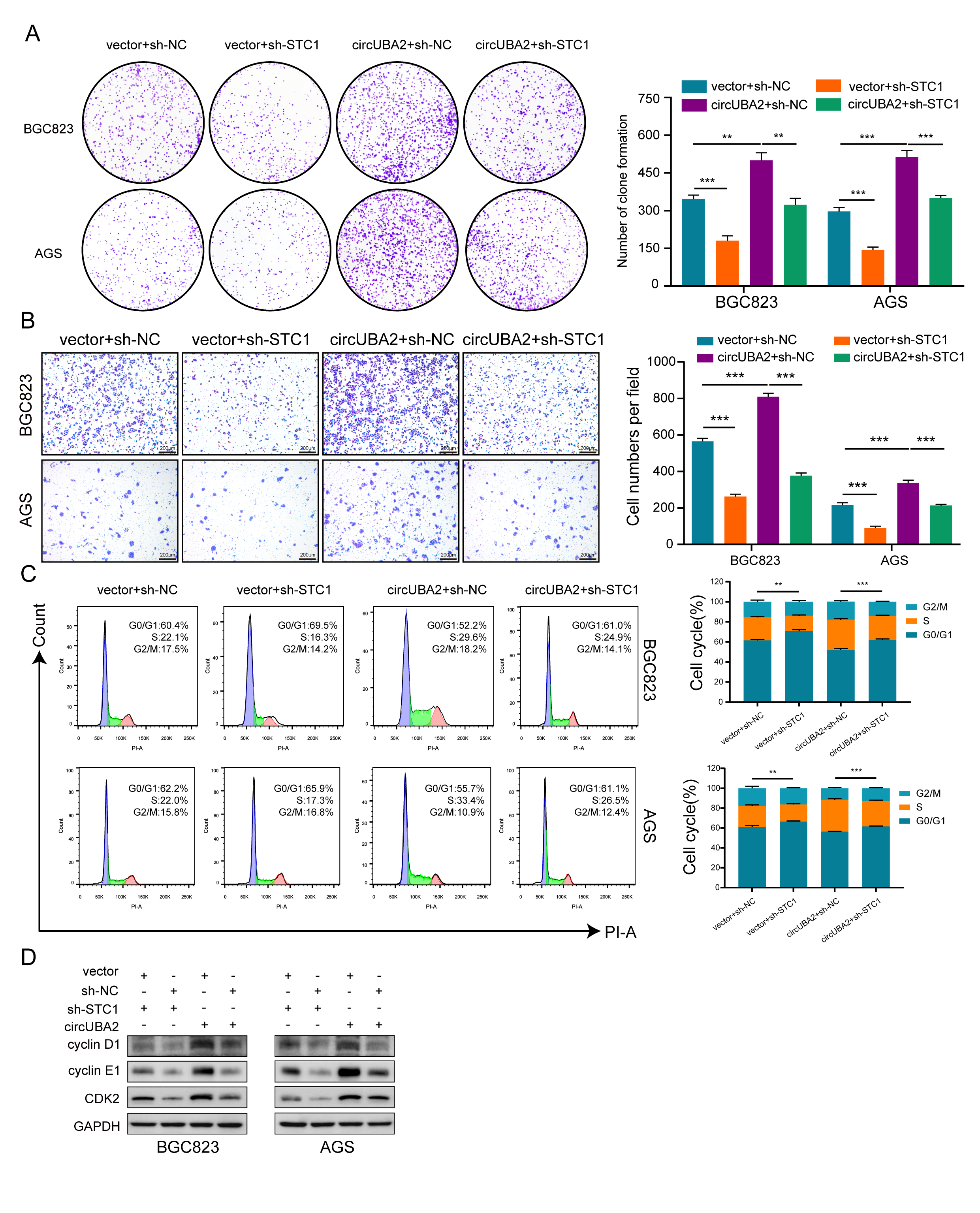

Supplement: Supplementary file 13 — Additional file 13. Figure S11: STC1 expression is upregulated in GC and correlates with poor prognosis. (A) Representative images and quantification of clone formation. (B) Representative images and quantification of migrated cells among indicated cells tested by transwell assay, scale bar = 200 μm. (C) Representative images of cell cycle distribution among indicated cells detected by flow cytometry. (D) Western blotting of cyclin proteins related to G1/S transition, including cyclin D1, cyclin E1, and CDK2 in transfected cells. *p <0.05; **p <0.01; ***p <0.001. [file 12935_2024_3423_MOESM13_ESM.tif]

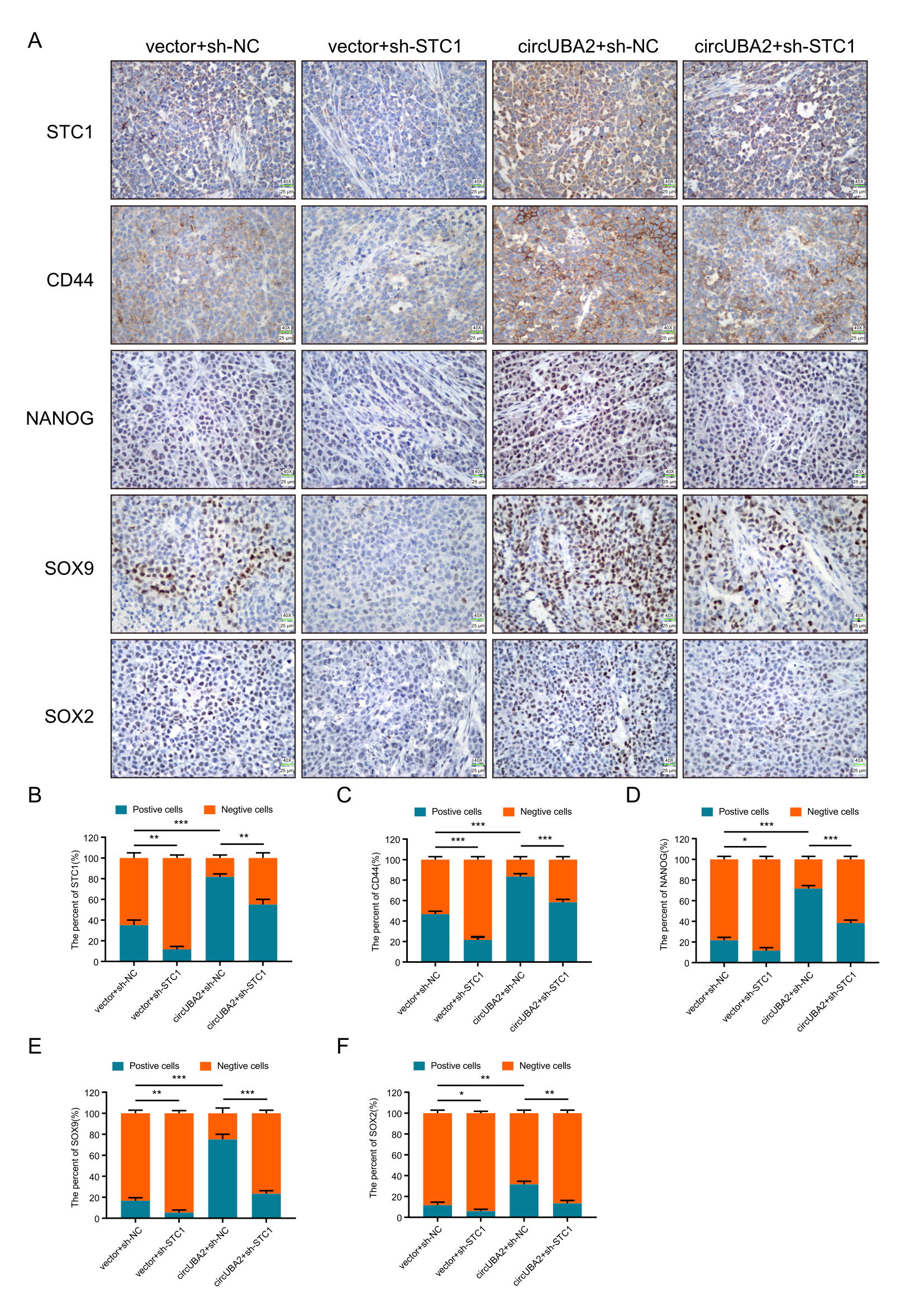

Supplement: Supplementary file 14 — Additional file 14. Figure S12: The expression of CD44, NANOG, SOX2, and SOX9 in xenograft tumours was detected by IHC. (A) STC1, CD44, NANOG, SOX9, and SOX2 IHC assays were adapted to detect the sections of nude mouse xenograft tumours injected with the indicated cells , scale bar = 25 μm. (B-F) The results of STC1, CD44, NANOG, SOX9 and SOX2 IHC were quantified. *p <0.05; **p <0.01; ***p <0.001. [file 12935_2024_3423_MOESM14_ESM.tif]

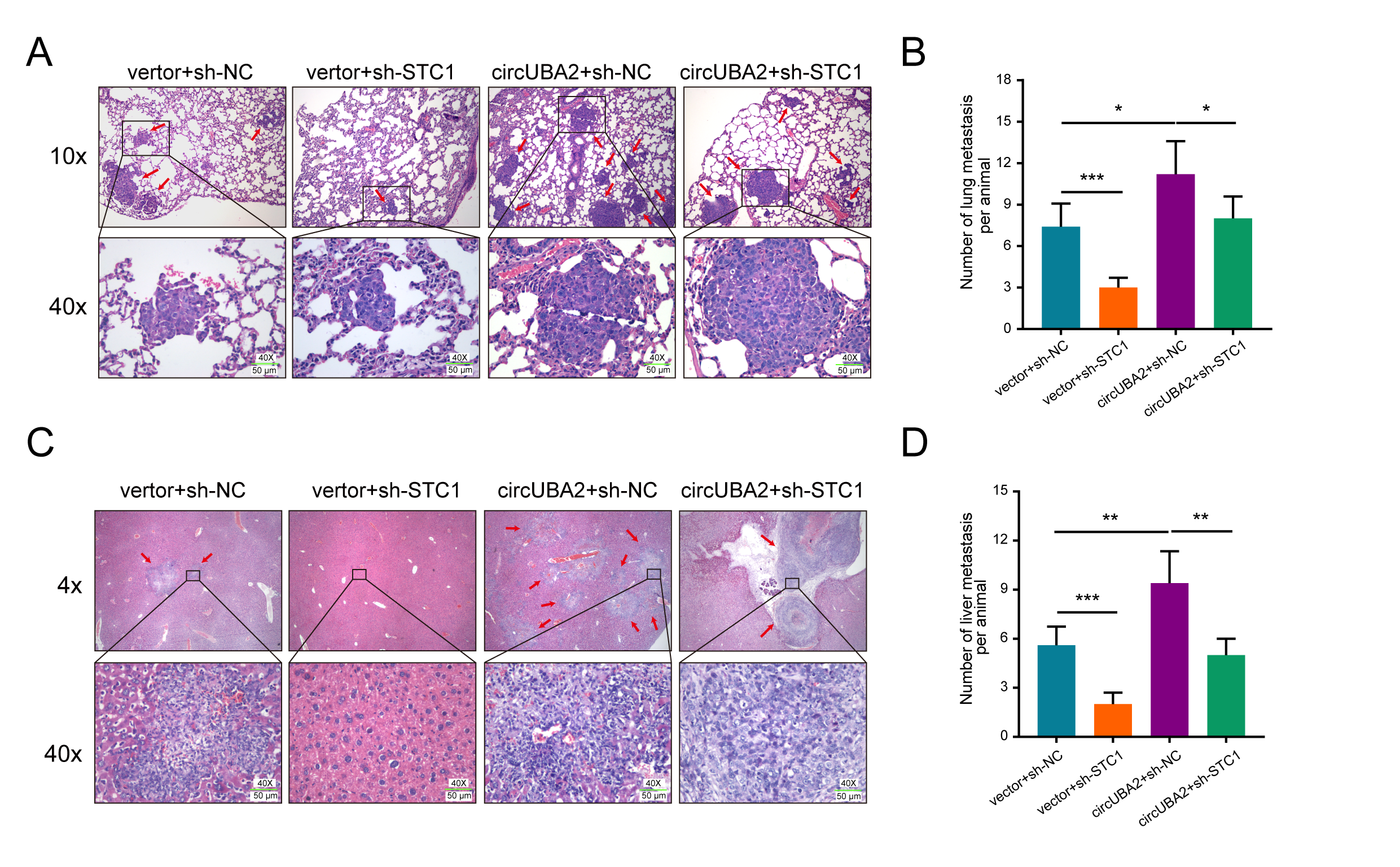

Supplement: Supplementary file 15 — Additional file 15. Figure S13: The rescue experiment associated with circUBA2 and STC1 in mice. (A) Representative images of HE stained lung tissues (n=5 mice per group), scale bar = 50 μm. (B) Quantification of lung metastases in mice injected with the indicated cells. (C) Representative images of HE stained liver tissue (n=5 mice per group), scale bar = 50 μm. (D) Quantification of liver metastases in mice injected with the indicated cells. *p <0.05; **p <0.01; ***p <0.001. [file 12935_2024_3423_MOESM15_ESM.tif]

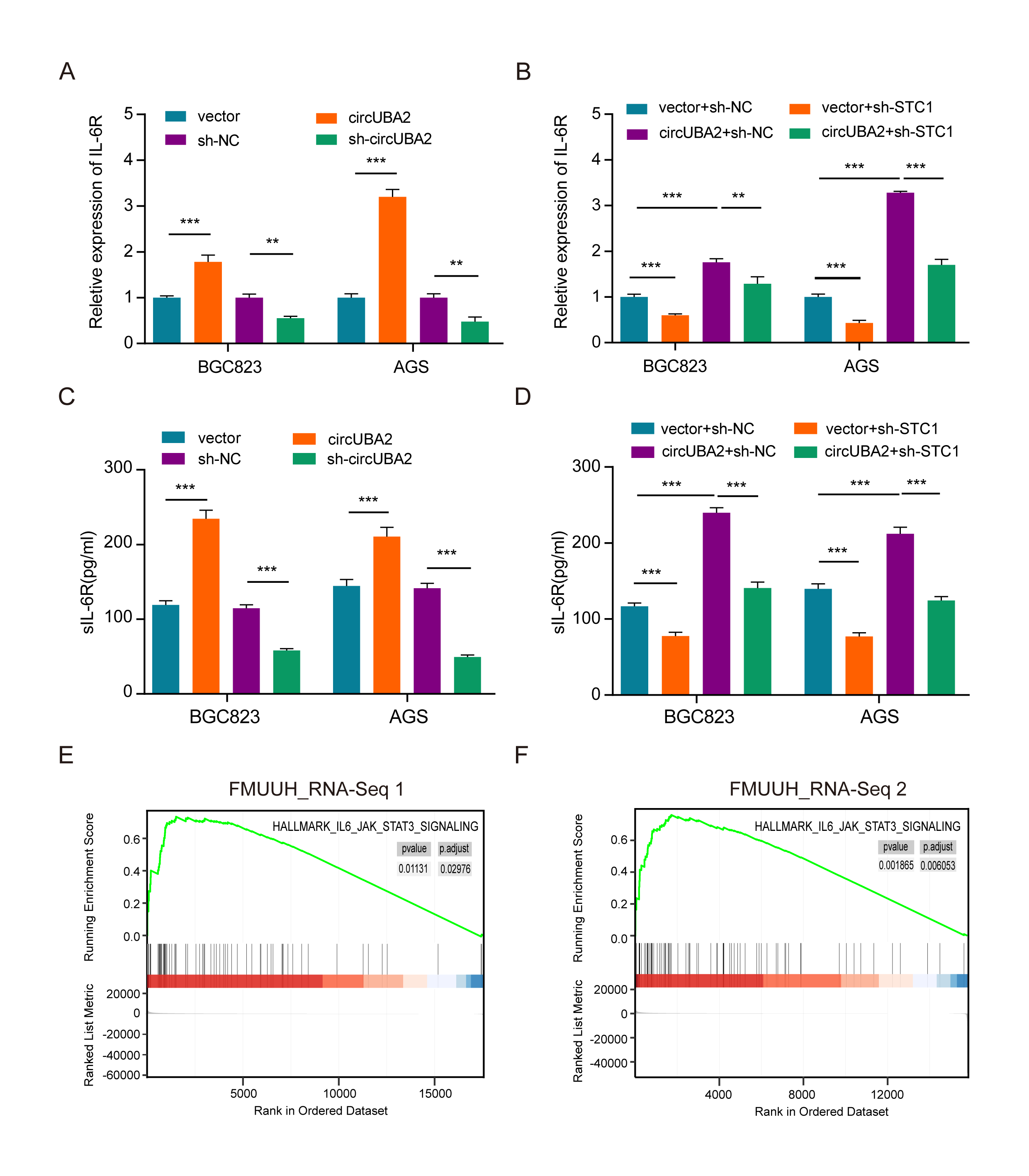

Supplement: Supplementary file 16 — Additional file 16. Figure S14: The expression of IL-6R, sIL-6R in GC cells and the GSEA of STC1-related pathway in our centre. (A-B) IL-6R expression in stably transfected BGC823 and AGS cells was determined by qRT-PCR. (C-D) sIL-6R expression in the supernatant of BGC823 and AGS cell was determined by ELISA. (E) GSEA of FMUUH_RNA-Seq 1 revealed a notable relationship between STC1 and ‘HALLMARK_IL6_JAK_STAT3_SIGNALING’ signaling pathway (n=60, p = 0.01131). (F) GSEA of FMUUH_RNA-Seq 2 revealed a notable relationship between STC1 and ‘HALLMARK_IL6_JAK_STAT3_SIGNALING’ signaling pathway (n=60, p = 0.001865). *p <0.05; **p <0.01; ***p <0.001. [file 12935_2024_3423_MOESM16_ESM.tif]
